# Physicochemically-guided immunomodulatory biomaterials for regulating immune responses in rheumatoid arthritis

**DOI:** 10.1016/j.mtbio.2026.103656

**Published:** 2026-09-07

**Authors:** Rui Sun, Chen Yue, Ning Yang, Ruoping Yanzhang, Peng Xiao, Youwen Liu, Xiaoqing Wang, Yiting Lei, Xiaolong Wu, Xue Zhang

**Affiliations:** a Henan Provincial Health Commission Key Laboratory of Bone Metabolism and Analysis, Luoyang Orthopedic-Traumatological Hospital of Henan Province(Henan Provincial Orthopedic Hospital), Zhengzhou, China; b Department of Rheumatology, Luoyang Orthopedic-Traumatological Hospital of Henan Province (Henan Provincial Orthopedic Hospital), Luoyang, China; c Department of Orthopaedic Surgery, The First Affiliated Hospital of Chongqing Medical University, Chongqing, China; d Chongqing Municipal Health Commission Key Laboratory of Musculoskeletal Regeneration and Translational Medicine, The First Affiliated Hospital of Chongqing Medical University, Chongqing, China; e Orthopaedic Research Laboratory of Chongqing Medical University, Chongqing Medical University, Chongqing, China; f Department of Biomedical Engineering, The Chinese University of Hong Kong, NT, China

**Keywords:** Immunomodulation, Biomaterials, Physicochemical properties, Rheumatoid arthritis

## Abstract

Rheumatoid arthritis (RA) is a chronic autoimmune disease that severely impairs the quality of life of patients owing to its complex immunopathological mechanisms and persistent joint damage. In recent years, the application of biomaterials in regulating immune responses in RA has attracted extensive attention, particularly regarding the regulatory effects of their physicochemical properties, including surface chemistry, mechanical properties, and morphology, on immune cell behavior and the inflammatory microenvironment. Current studies have revealed that rationally designed biomaterials can modulate inflammatory responses by influencing the activation, differentiation, and signaling pathways of immune cells, thereby providing novel insights into RA treatment. However, the specific regulatory mechanisms underlying different physicochemical properties remain incompletely elucidated, and related research still faces challenges related to material biocompatibility and immune specificity. This article systematically reviews the latest advances in the research of biomaterial physicochemical properties in the field of RA immunomodulation, analyzes the mechanisms of their action in regulating the immune microenvironment, and discusses the potential and future directions of biomaterial-based immunomodulatory strategies in clinical applications to provide theoretical basis and technical support for the development of precision immunotherapy.

## Graphical abstract

This review explores how biomaterial surface chemistry, matrix stiffness, and morphological structure influence immune cell behavior and the inflammatory microenvironment. It analyzes the design strategies and applications of Rheumatoid Arthritis (RA) immunomodulatory biomaterials based on physicochemical properties. Furthermore, it highlights that current research has shifted from a single anti-inflammatory approach to a synergistic regulation integrating immune response, metabolism, and bone/cartilage regeneration. Despite significant progress, current studies still face challenges in multi-parameter synergistic effects, regulatory molecular mechanisms, and complex in vivo models. This review emphasizes that the rational design of physicochemical parameters of biomaterials is pivotal for achieving active immunomodulation and provides an outlook on future research directions in this field.

**Figure:**
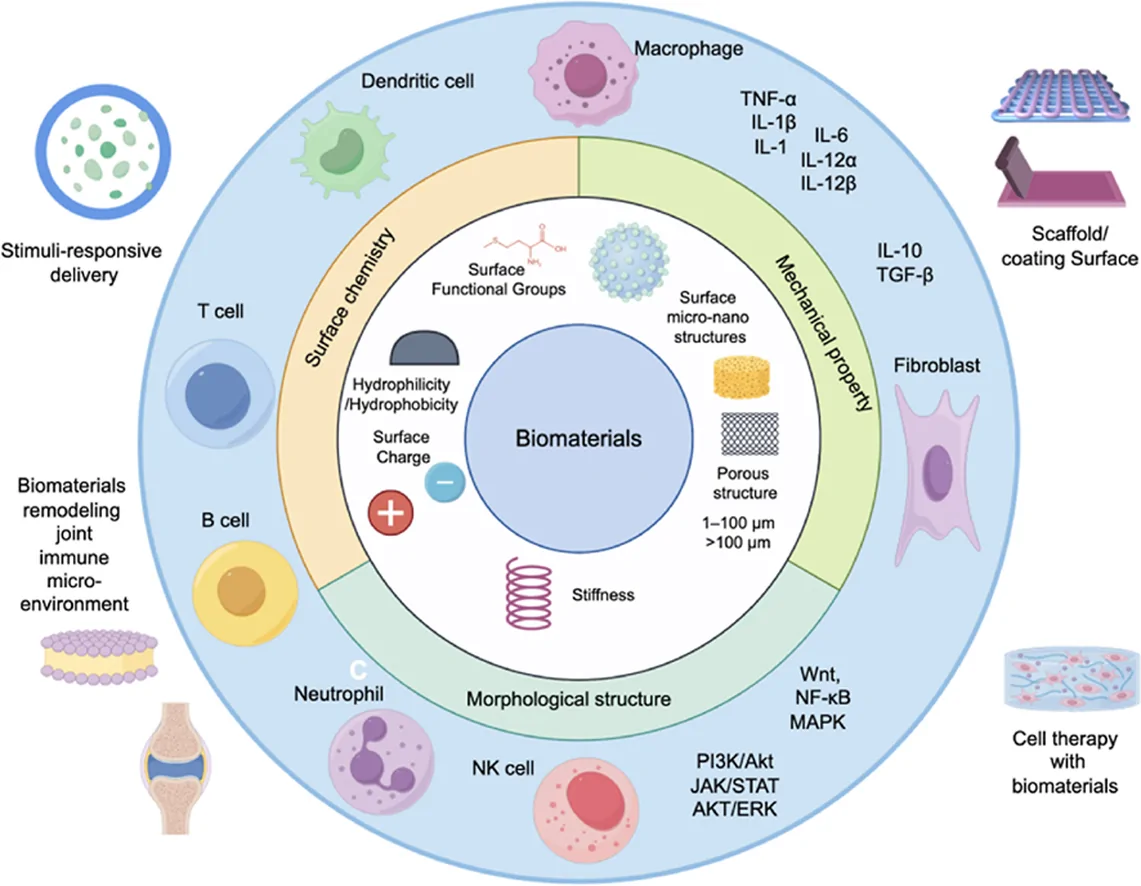

## 1 Introduction

### 1.1 Pathogenesis and treatment of rheumatoid arthritis (RA)

RA is a common systemic autoimmune disease with a complex pathogenesis [[1], [2], [3]]. Its primary manifestations include synovial inflammation and joint destruction, causing joint pain, deformity, and limited mobility in patients, significantly affecting their quality of life [[4], [5], [6]]. Global epidemiological data shows that the incidence of RA ranges from 0.5 to 1.0% [7], with the incidence rate in women being two-to three-fold higher than in men [8]. RA is more common in the elderly population aged 65–69 years [9]. The pathogenesis of RA involves genetic susceptibility, environmental factors, and abnormal regulation of the immune system, which manifests as the abnormal activation of immune cells and imbalance of inflammatory factors, resulting in a persistent chronic inflammatory state [10,11]. Although the specific composition and function of innate immunity is still undergoing study, its interaction with the adaptive immune system is of crucial importance for disease regulation [12]. Notably, studies have shown that the interaction between innate immunity and the adaptive immune system is crucial for disease regulation [13].

The traditional treatment methods for RA primarily include non-steroidal anti-inflammatory drugs, glucocorticoids, disease-modifying antirheumatic drugs, and biological agents [[14], [15], [16]]. Although these methods can alleviate symptoms and slow the progression of the disease, they have limitations such as limited efficacy, significant side effects, and significant individual differences between patients [17,18]. The immunological heterogeneity of RA and the diversity of the disease pose challenges for precise treatment, and more personalized treatment strategies are required [17]. In recent years, with advances in the understanding of the mechanism of RA, immunomodulation has become a topic of interest in therapeutic research, especially in regulating the functions of immune cells and balancing the levels of inflammatory factors [13,17].

### 1.2 Biomaterials as immunomodulatory tools

The abnormal activation of immune cells and imbalance of inflammatory factors are central to RA [19]. Traditional drugs rely on a single molecular target and exhibit significant systemic toxicity, making it difficult to precisely regulate local immunity. Therefore, searching for new methods that can precisely regulate the local immune microenvironment of the joints has become a significant research interest [20]. Owing to their controllable physicochemical properties and excellent biocompatibility, biomaterials have become an important tool for regulating RA immune responses and improving the inflammatory state [21,22].

Biological materials can directly modulate immune cell behavior at the cell–material interface through physicochemical signals, including surface charge, stiffness, and morphology [23,24]. The key advantages of this approach include local enrichment with sustained effects, reduced systemic toxicity, direct regulation through physical signals, synergistic drug/photothermal enhancement, tunable physicochemical properties to accommodate individual differences, and multi-target synergy and remodeling of the immune network [25]. Based on this, we propose the core hypothesis that the synergistic design of material charge, stiffness, and morphology can simultaneously regulate macrophage M1/M2 polarization, the T helper (Th)17/T regulatory (Treg) cell balance, and fibroblast-like synoviocyte (FLS) activation within the same joint microenvironment of RA, thereby interrupting the positive feedback loop of inflammation and re-establishing immune homeostasis.

The above core assumption has been preliminarily verified. For instance, the methotrexate microsponge (MTX-MSP) with its unique viscoelastic properties ensures injectability as well as enables long-term retention and continuous release of the drug within the joint cavity, thereby effectively reducing inflammation and minimizing systemic toxicity [26]. After being treated with oxygen plasma, the alginate-tyramine hydrogels (ALG-TYR) can directly regulate the behavior of immune cells through its surface chemical properties, thereby achieving immunomodulation [27]. Moreover, injectable thermosensitive hydrogels can undergo reversible sol-gel conversion at physiological temperatures [28]. These gels have been used for the continuous delivery of biological agents such as tumor necrosis factor-α (TNF-α) inhibitors into the joint cavity, serving as a convenient carrier for precise treatment [29]. These examples reveal that the behavior of key cells in the joint immune microenvironment can be influenced by appropriately designing the physicochemical properties of biomaterials.

### 1.3 Biomaterials regulate key immune cells in RA

Within the RA microenvironment, macrophages, T cells, dendritic cells (DCs), and FLSs engage in complex pathological interactions largely mediated by secreted cytokines and inflammatory mediators [30]. The balance of M1/M2 polarization is critical for controlling RA progression [31]. M1 macrophages release pro-inflammatory cytokines such as TNF-α, interleukin (IL)-1β, and IL-6, which exacerbate joint inflammation. In contrast, M2 macrophages secrete anti-inflammatory cytokines such as IL-10 and TGF-β, which suppress inflammation and promote tissue repair [32]. Macrophage-derived cytokines influence T-cell differentiation, leading to an imbalance between Th17 and Treg cells, which play important roles in RA inflammation [33,34]. As antigen-presenting cells (APCs), DCs promote Th17 differentiation by presenting self-antigens. Therefore, modulating DC function through drug-loaded biomaterials can regulate the Th17/Treg balance upstream and promote the resolution of inflammation [35]. The cytokines released by T cells, macrophages, and DCs act synergistically with FLSs. As mesenchymal cells, FLSs inherently possess invasive, migratory, matrix-remodeling, and intercellular communication capabilities. In RA, FLSs secrete inflammatory cytokines and matrix metalloproteinases, which directly promote inflammation and synovial hyperplasia [36]. In addition, they recruit additional immune cells through chemokines, thereby amplifying the inflammatory response. The transcriptional features of specific FLS subpopulations are also cooperatively regulated by T cells and macrophages [37]. Given the complex interactions and polarization imbalances among macrophages, T cells, DCs and FLSs, biomaterials can serve as exogenous interventions. Through physicochemical properties such as surface modification, nanostructures or drug loading, they can synergistically regulate M1/M2 polarization, Th17/Treg balance, DC function, and FLS activation, thereby balancing inflammatory cytokines and achieving immunomodulation.

In summary, the pathogenesis of RA comprises an abnormal activation of immune cells and an imbalance of inflammatory factors. However, traditional treatments have several limitations in terms of accuracy and safety. Owing to their highly customizable physical and chemical properties, biomaterials have become a tool for the regulation of the immune microenvironment of joints with RA and for precise immune regulation. Studying the regulatory relationship between the physical and chemical properties of materials and the behavior of critical immune cells can assist in the development of efficient, low-toxicity and individualized treatment strategies. In the future, the treatment of RA will shift from alleviating symptoms to achieving immune homeostasis with the integration of materials science and immunology.

## 2 Surface chemistry of biomaterials in immunomodulation

Surface chemistry encompasses surface functional groups, hydrophilicity or hydrophobicity, and surface charge. It regulates immune responses by influencing the recognition, adhesion, and activation of immune cells.

### 2.1 Surface functional groups modulate immune recognition and microenvironment responsiveness

Surface functional groups significantly influence immune cell recognition. The surface functional groups of biomaterials regulate the functions of immune cells such as recognition, adhesion, and polarization, as well as their secretion of inflammatory factors and phagocytosis. Di-hydroxyl modified cationic nanoparticles (cNPs) minimize opsonin adsorption (such as IgM and complement), prolonging circulation and enhancing inflamed-joint retention. They electrostatically scavenge cell-free DNA (cfDNA) from dying cells, preventing Toll-like receptor 9 (TLR9) binding and NF-κB activation, thus reducing macrophage pro-inflammatory cytokine secretion of TNF-α, IL-6, and IL-1β, and halting the intra-articular inflammatory cascade at its source [38]. Surface chemistry directly affects the expression of TLRs in macrophages. Surfaces rich in hydrocarbons increase the expression of TLR2 and upregulate pro-inflammatory genes, whereas surfaces rich in carboxyl, amino, and oxazoline functional groups enhance the expression of TLR4 and upregulate anti-inflammatory genes such as IL-10 [39].

**Fig. 1:**
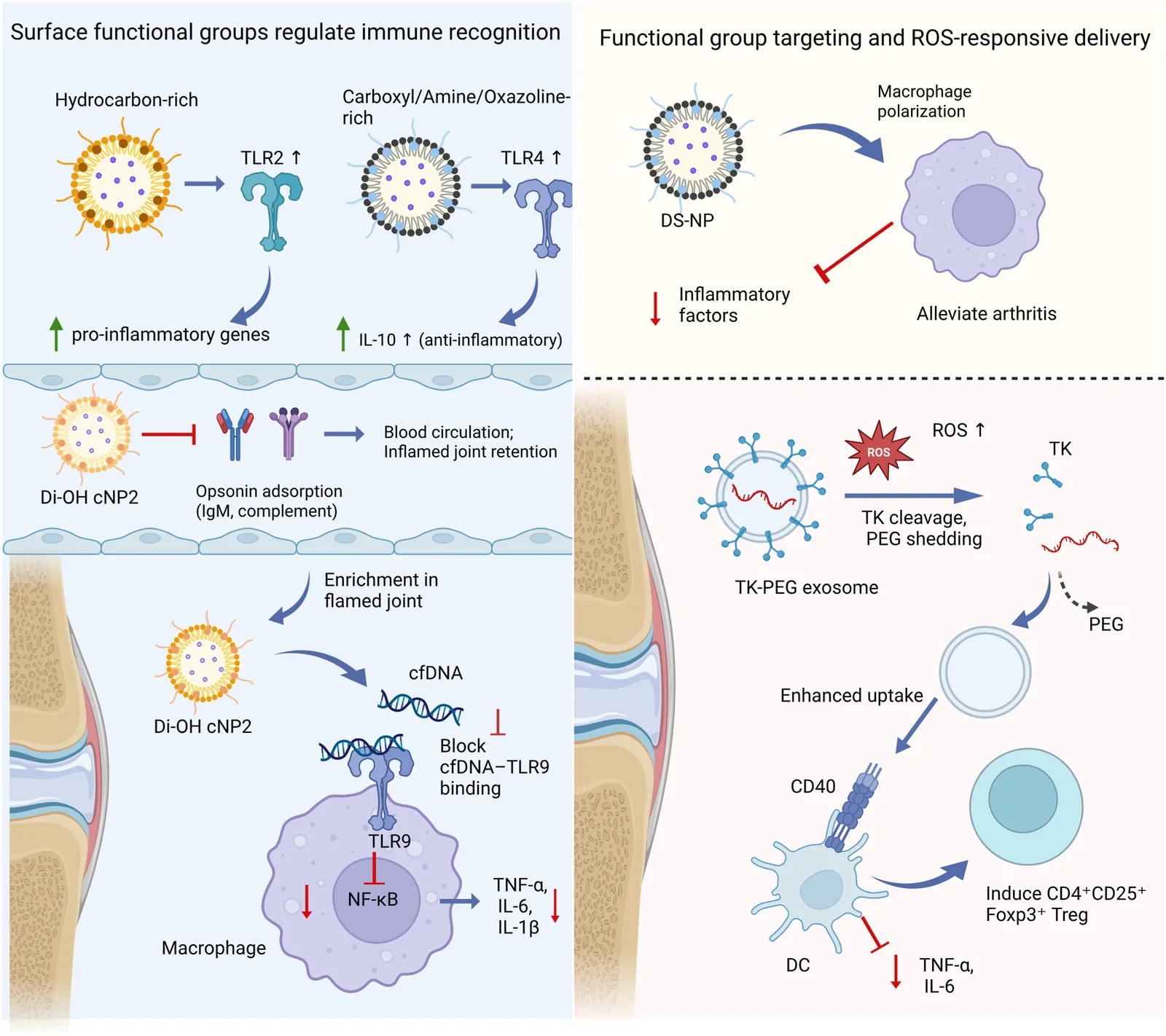
Chemical mechanism of the surface functional groups of biomaterials. Hydroxyl-rich cNP2 extends circulation, targets inflamed joints, scavenges cfDNA, and blocks TLR9/NF-κB signaling, thereby reducing TNF-α/IL-6/IL-1β. Hydrocarbon-rich surfaces upregulate TLR2, pro-inflammatory genes; carboxyl/amine/oxazoline-rich surfaces upregulate TLR4 and IL-10; and DS-NPs target synovial macrophages, promote anti-inflammatory polarization, and relieve arthritis. TK-PEG exosomes respond to ROS by shedding PEG, enhancing DC uptake, reducing CD40 and TNF-α/IL-6, and inducing Tregs to restore immune tolerance.

Functional group-specific targeting of macrophages and reactive oxygen species (ROS)-responsive drug delivery represent two complementary strategies for achieving precise immunomodulation. Nanoparticles modified with dextran sulfate (DS) use their surface sulfate groups for specific targeting of macrophages in the RA synovium, enhancing the induction of macrophage transformation by the drug, inhibiting the release of inflammatory factors, and alleviating arthritis [40]. Lee et al. introduced TK-PEG-modified DC exosomes into RA lesions. ROS triggered TK cleavage and PEG detachment, promoting the efficient uptake of exosomes by DCs and down-regulating CD40, inhibiting TNF-α and IL-6 secretion, inducing CD4^+^CD25^+^Foxp3^+^ Treg differentiation, and ultimately breaking the immune imbalance and promoting immune tolerance [41]. As ROS serve as central signaling molecules governing cellular redox homeostasis [42], biological materials actively regulate immune cells through surface-modified functional groups and modulate the secretion of inflammatory factors; they achieve targeted delivery or use ROS responses to deliver drugs and achieve local administration to reduce systemic toxicity (Fig. 1).

However, most studies comprise in vitro or concept validation, resulting in a simplified association between surface functional groups and TLRs, without exploring the complexity and overlap of the network signaling pathways in RA inflammation. Functional groups often exhibit surface charges and hydrophilicity, making it difficult to attribute them to a single cause independently. In addition, there is a lack of data regarding the protein corona effect, toxicity, and long-term safety in vivo.

### 2.2 Hydrophilic-hydrophobic design for responsive release and immunomodulation

**Fig. 2:**
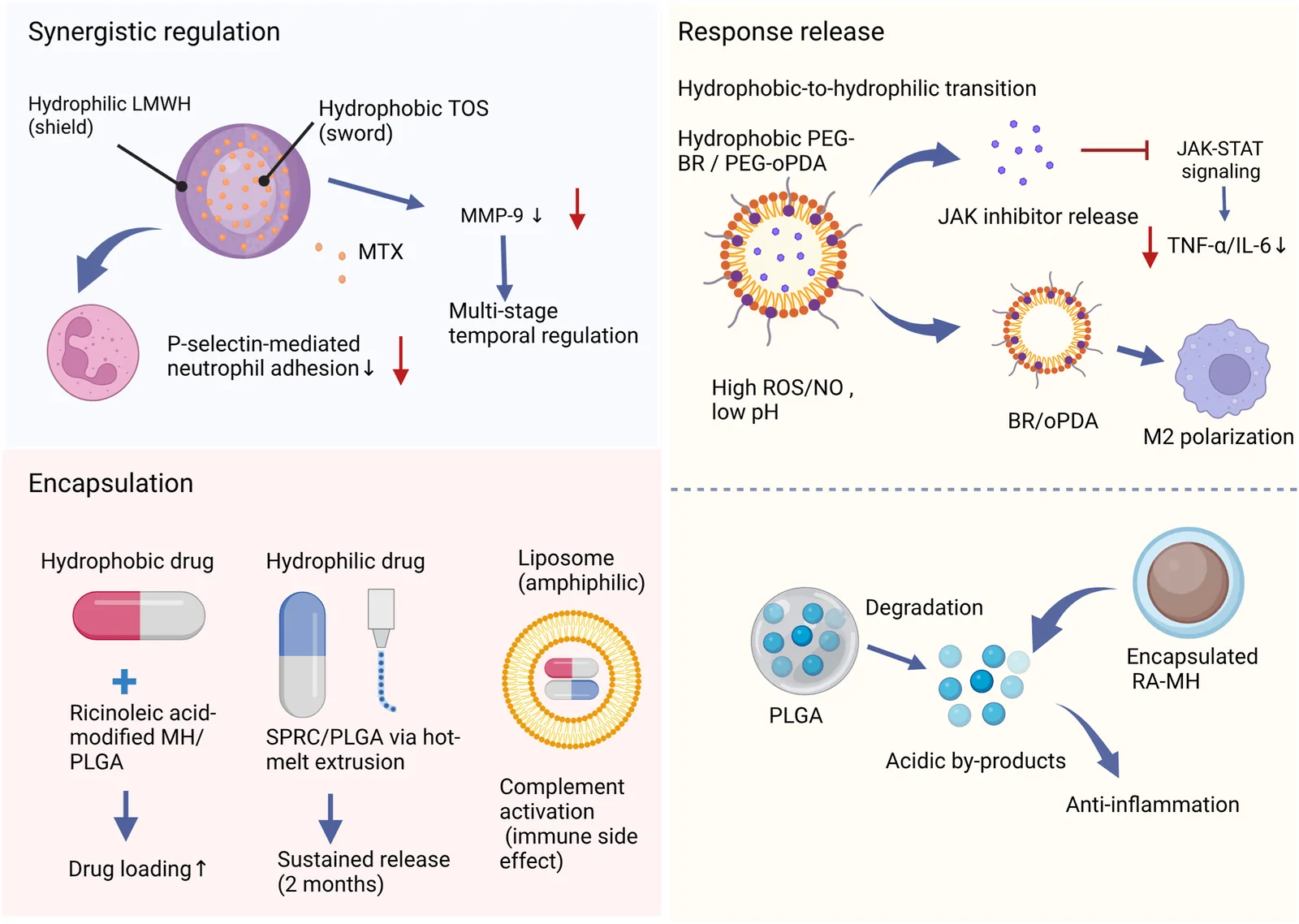
Chemical mechanism of the hydrophilicity and hydrophobicity of biomaterials. Hydrophilic LMWH (shield) and hydrophobic TOS (sword) inhibit neutrophil adhesion and MMP-9; ROS/NO-triggered disintegration releases JAK inhibitors, scavenges ROS/NO, and promotes M2 polarization; and Mg(OH)_2_ neutralizes acidic PLGA by-products to suppress TNF-α/IL-6. Hydrophobic/hydrophilic drug encapsulation strategies achieve sustained release and targeting, although complement activation risk is present.

Hydrophilicity and hydrophobicity of biomaterial surfaces are critical parameters for constructing drug delivery systems and regulating pathological microenvironments. They directly affect the assembly of nanocarriers, targeting efficiency, drug release dynamics, and interactions between immune cells (Fig. 2).

Hydrophobic and hydrophilic properties of nanoparticles enable multi-stage sequential regulation. Li et al. determined that the hydrophilic low-molecular-weight heparin (LMWH) antagonizes the neutrophil adhesion mediated by P-selectin, similar to a shield, whereas the hydrophobic TOS downregulates the expression of matrix metalloproteinase 9 (MMP-9), similar to a sword, achieving multi-stage temporal regulation [43].

The RA immune microenvironment exhibits high levels of ROS/nitric oxide (NO) and low pH features, which trigger the hydrophobic-hydrophilic phase transition of the nanomaterial, thus enabling precise in-situ controlled release at the inflammatory site. The hydrophobic structures of PEG-BR and PEG-oPDA transform into hydrophilic structures under the influence of ROS/NO, undergo oxidation and disintegration, and release JAK inhibitors. The loaded drug inhibits the JAK-STAT signaling pathway, thereby reducing the secretion of pro-inflammatory cytokines and chemokines. The hydrophobic or hydrophilic structures of BR and oPDA eliminate ROS and NO, synergistically promoting M2 polarization, reducing immune cell recruitment, and thereby breaking the positive feedback loop of inflammation [44]. PLGA degradation produces acidic by-products, resulting in a decrease in local pH [45]. By encapsulating magnesium hydroxide (RA-MH) particles with hydrophobic modification, the acidic by-products are neutralized, inhibiting the release of pro-inflammatory cytokines TNF-α and IL-6 and thereby exerting an anti-inflammatory effect [46].

The hydrophilicity and hydrophobicity of biomaterials affect the encapsulation and sustained release behavior of RA therapeutic drugs and may also cause immune side effects. Regarding hydrophobic drugs, after modifying MH with ricinoleic acid and encapsulating it into PLGA, the drug loading capacity is increased [46]. For highly hydrophilic drugs, the hot-melt extrusion technique embeds SPRC into the PLGA matrix, extending the sustained-release period to 2 months [47]. In contrast, liposomes are amphiphilic as classical nanocarriers. They simultaneously encapsulate hydrophilic and hydrophobic drugs and deliver the drugs to the lesion through passive or active targeting, but can cause immune side effects such as complement activation [48].

The research on hydrophilic and hydrophobic structures primarily focuses on drug delivery systems [49], targeted macrophages [50], pH/NO responsive release [51,52], improvement of joint lubrication effect [53], and indirect enhancement of immune regulatory function.

In summary, the hydrophilicity and hydrophobicity of biomaterial surfaces have transcended their basic physical and chemical properties and have become core elements in the design of complex functional systems. By constructing multifunctional nanoparticles with both hydrophilic and hydrophobic properties [43], developing intelligent conversion materials responsive to pathological microenvironments [54], and optimizing targeted delivery and sustained-release systems based on hydrophilic-hydrophobic interactions [47,48,50], multi-dimensional and sequential regulation of the inflammatory microenvironment and immune cells can be achieved. However, the hydrophobic/hydrophilic structure designed outside the body cannot be covered by the protein shell inside the body, and the therapeutic effect is uncontrollable. Notably, the ROS/NO response design is irreversible, and the function of the material disappears after drug release. Its single-time clearance ability is limited. Although the side effect of complement activation by liposomes has been considered, the hypersensitivity reaction caused by it and the subsequent decline in therapeutic effect have not been extensively addressed. The hydrophobic and hydrophilic properties, functional groups, particle sizes, and other characteristics interact with each other. Therefore, in future studies, a multi-factor design model should be considered.

### 2.3 Surface charge modulation of protein adsorption and immune cell responses

Surface charge directly regulates the inflammatory response in RA by affecting protein adsorption and immune cell attachment. Studies have shown that the surface charge zeta potential of biomaterials is a key factor determining their interaction with biological fluids [55]. Surfaces with positive charges adsorb more proteins and promote the adhesion of platelets and white blood cells [56]. When the surface charge zeta potential ranges −10 to +10 mV, and has a highly hydrophilic interface, the blood compatibility is good, reducing non-specific protein adsorption and blood cell adhesion and alleviating inflammation [56]. This indicates that in the treatment of RA, designing biomaterials with neutral or moderately negative charge is beneficial for creating an anti-inflammatory microenvironment.

The surface charge affects the behavior of macrophages, with an M1/M2 polarization balance that is crucial in RA progression. It has been shown that molecular mobility and the zeta potential of biomaterial surfaces independently regulate the response sequence of macrophages. A more negative zeta potential can inhibit the initial spreading of macrophages [57]. In addition, differences in surface charges, such as the shift in isoelectric point IEP, reflect changes in the surface functional groups of the material [55], thereby affecting cell recognition and response.

**Fig. 3:**
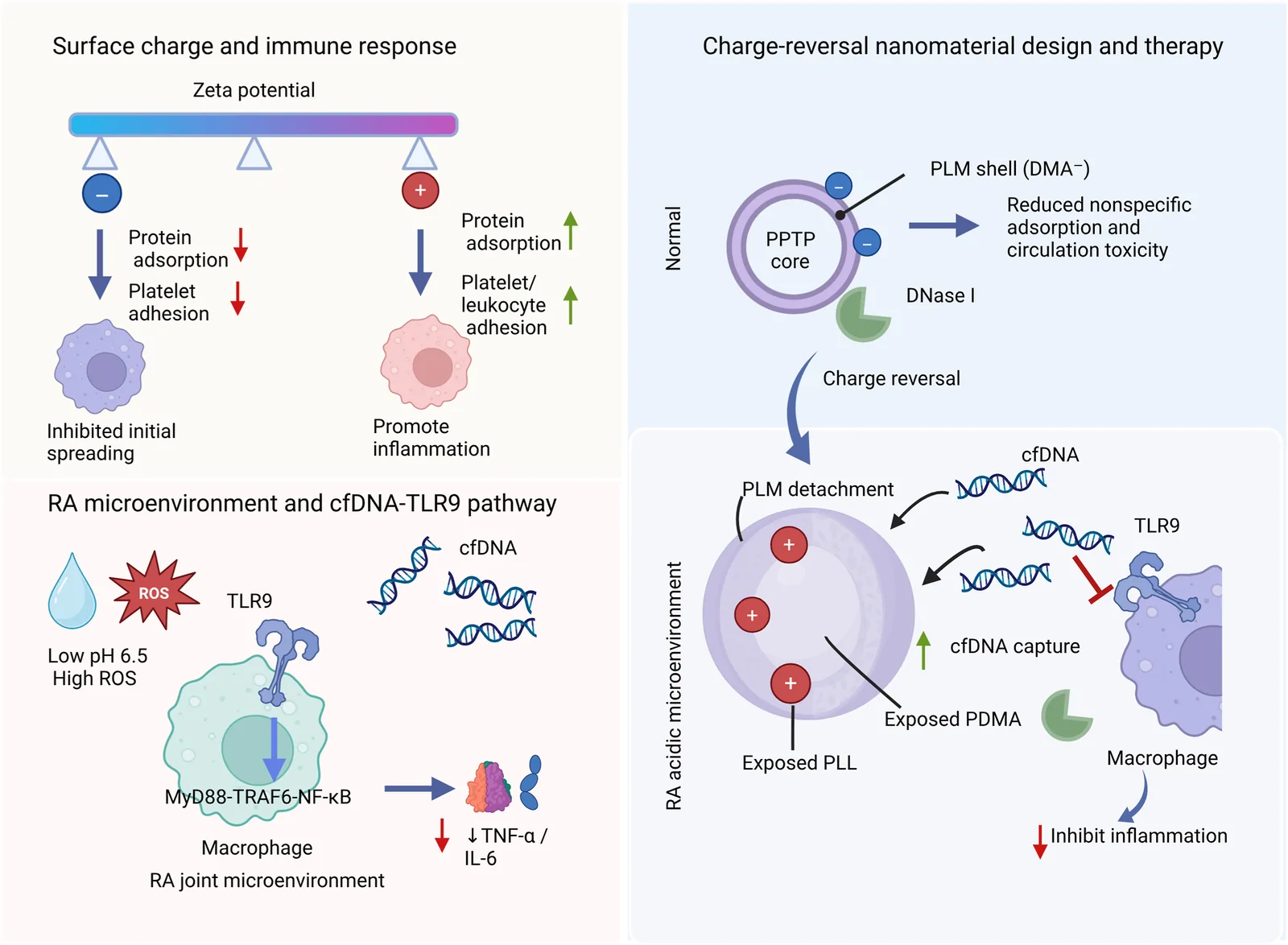
Chemical mechanism of the surface charge of biomaterials. Positive surfaces increase protein adsorption, platelet/leukocyte adhesion, and M1 polarization, whereas negative/neutral surfaces (zeta −10 to +10 mV) reduce nonspecific protein adsorption and promote M2 polarization. In the RA microenvironment (pH 6.5, high ROS), the charge-reversal nanogel (PLM/DMA shell, PPTP core, and DNase I) detaches the negative shell, exposes PLL/PDMA, captures/degrades cfDNA, blocks TLR9-MyD88-TRAF6- NF-κB signaling, and decreases TNF-α/IL-6.

From the molecular mechanism point of view, the surface charge affects the subsequent cell fate by altering the electrostatic interaction between the material and the protein. Protein adsorption on the surface of biomaterials is an important process for cell response [58]. Notably, the measurement of zeta potential and isoelectric point is an effective tool for evaluating surface charges and protein adsorption behavior [55,57]. The regulation of surface charges provides a novel avenue for targeted treatment of RA. During RA pathogenesis, cfDNA within the joint cavity is a key pathogenic factor. Preventing the binding of cfDNA to TLR9 and inhibiting inflammation have become a major therapeutic strategy. The DG3K10 nanogels capture pathogenic cfDNA and use surface-conjugated DNase I to degrade it, thereby down-regulating TLR9 activation and suppressing the downstream MyD88-TRAF6-NF-κB-dependent cascade. This in turn inhibits the expression of pro-inflammatory cytokines such as TNF-α and IL-6 [59]. However, when overly strong positive charges circulate within the body, they tend to adsorb plasma proteins, forming protein caps. This reduces the efficiency of cfDNA capture and may also trigger cytotoxicity. Therefore, for the acidic microenvironment of joints with RA (pH = 6.5) and the protein environment, nanomaterials with charge reversal properties have been designed [60,61]. These materials are composed of a core PPTP and outer shell PLM. Under normal physiological conditions, the PLM layer provides negative charge shielding through DMA groups, making the nanoparticles negatively charged overall and reducing non-specific protein adsorption and circulation toxicity. When reaching the acidic microenvironment of joints with RA, the DMA groups hydrolyze and detach, causing the PLM layer to undergo charge reversal and detach and exposing positively charged PLL. Simultaneously, the thioketone bonds in the core PPTP break under high ROS levels, further exposing the strongly positively charged PDMA, thereby significantly enhancing the electrostatic capture ability for cfDNA [61]. Charge-reversal materials enhance cfDNA capture and degradation in the RA microenvironment, block TLR9, and inhibit inflammation (Fig. 3).

Recent studies have shown that negative charges promote anti-inflammatory effects, whereas positive charges are used for targeted delivery or clearance of cfDNA. These seemingly contradictory conclusions result from the differences in the internal and external environmental conditions. The zeta potential in vitro is modified or shielded by protein caps in vivo, and it may not inhibit macrophage spreading. The particle size, charge density, hydrophilic functional groups, and charge are coupled, making it easy to attribute the combined effect to the charge. The protein caps determine cell recognition and the original charge may be masked or reversed. It cannot be simply concluded that positive or negative charges are optimal. Negative charges reduce adhesion, maintain biocompatibility, and are suitable for the circulation interface, whereas positive charges actively target and are suitable for local treatment. Future research should focus on controlling variables and dynamic changes and conducting in vivo validation. Biological materials are designed to change their surface charges according to the environmental conditions. Therefore, they have good biocompatibility and can target inflammation, actively participating in immune regulation.

After hydrophilic chitosan is modified with hydrophobic groups and coupled with folic acid, it can target the folic acid receptors on macrophages and enhance internalization [62]. According to the co-design principle, negative charges are introduced, which may decrease the uptake efficiency owing to electrostatic repulsion. Therefore, the balance between hydrophobicity and charge is crucial in the design of targeted nanoparticles and should be optimized based on the receptor expression and membrane charge properties of the target cell. The hydrophilic surface tends to induce M2 polarization; however, the hydrophilic material with high stiffness (295 kPa) promotes M1 polarization. This reveals a strong connection between surface chemistry and mechanical properties. Optimizing only for chemical properties does not guarantee anti-inflammatory effects; stiffness must also be jointly regulated. Dynamic changes in the RA microenvironment require materials to shift from static to sensing-feedback designs, such as ROS/pH response reversals. This design is normal in the blood and activates the regulation of immunity upon entry into the synovium. The details of the activation of TLR4 by the sulfate group, the effects of the same hydroxyl modification on the conformations of the protein crowns under different nanocurvature conditions, and the mechanism of charge transfer to T cells and synovial fibroblasts should be explored in depth.

## 3 Role of the mechanical properties of biomaterials in RA immunomodulation

**Fig. 4:**
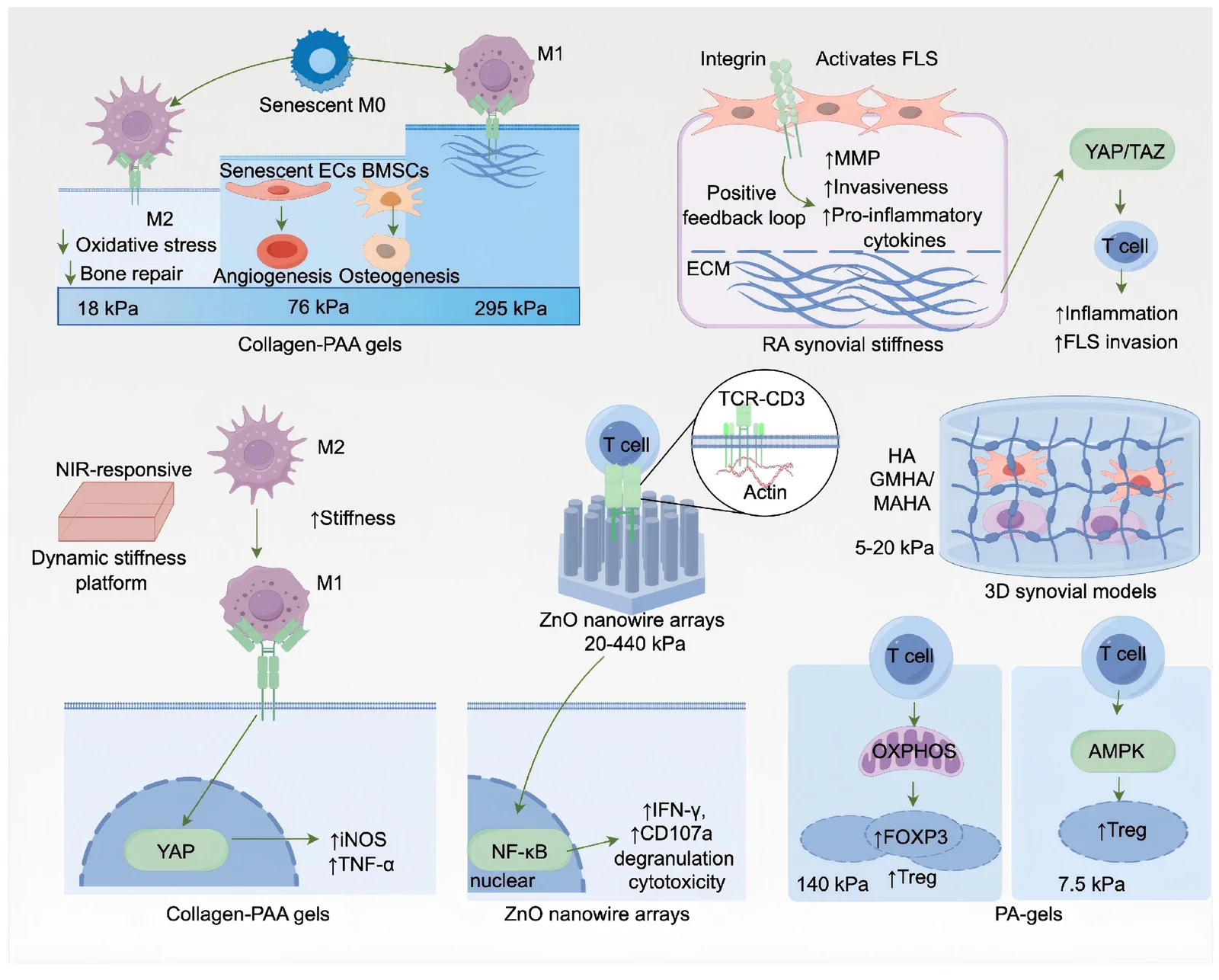
**Biomaterial stiffness affects immune cells in RA.** Stiff substrates promote M1 macrophages (pro-inflammatory), whereas soft substrates promote M2 macrophages (anti-inflammatory) and help tissue repair. In RA, increased synovial stiffness activates FLSs through integrins and YAP/TAZ, increasing MMP, Th17, and IL-6, and leading to synovial hyperplasia and bone erosion. Stiffness also affects T cells: hard gels enhance OXPHOS and support Treg formation. Hydrogels with stiffness similar to that of the RA synovium (5–20 kPa) provide 3D models to study FLS migration and immune cell behavior. The image was created using figdraw.com.

The pathogenesis of RA involves pathological remodeling of the joint synovial mechanical microenvironment. The mechanical properties include stiffness. Owing to its significance, we primarily explore the mechanism of its action. Biomaterial stiffness determines immune cell function through mechanotransduction pathways: high stiffness promotes M1 polarization, whereas low stiffness favors M2 polarization. In addition, rigidity modulates T cell activation thresholds, differentiation, and metabolic reprogramming, while also modulating the invasive phenotype of FLSs and pro-inflammatory cytokine secretion (Fig. 4).

The stiffness of the material affects the polarization state of the macrophage. It has been shown that materials with high stiffness promote proinflammatory M1 polarization, whereas materials with low stiffness induce M2 polarization. For instance, using a dynamic stiffness material platform with a near-infrared response, as the stiffness of the biomaterial increased, the expression of iNOS and TNF-α in bone marrow-derived macrophages (BMDMs) was upregulated, indicating that macrophages transformed from M2 to M1. Simultaneously, the increase in the proportion of nuclear YAP protein was positively correlated with the change in the polarization state of macrophages [63]. When using collagen-coated polyacrylamide hydrogels of different stiffness (18, 76, and 295 kPa), the high stiffness (295 kPa) promotes the polarization of senescent macrophages (S-MΦ) towards M1, whereas the decreased stiffness (18 and 76 kPa) promotes M2 polarization. M2 senescent macrophages, especially on the 76 kPa matrix, significantly enhance the angiogenesis and osteogenic differentiation of senescent endothelial cells and senescent bone marrow mesenchymal stem cells (S-BMSCs). This helps to alleviate cellular oxidative stress and promotes the repair and regeneration of aged bone tissue [64]. These findings reveal that the stiffness of the material affects the polarization of macrophages as well as indirectly promotes the tissue regeneration process by modulating the immune microenvironment. However, whether YAP is a necessary and sufficient condition for stiffness-driven polarization of macrophages remains to be verified.

The increased stiffness of synovial tissue in RA activates FLSs through integrin-mediated mechanotransduction. This activation enhances FLS invasiveness and MMP expression. Activated FLSs release large amounts of pro-inflammatory cytokines that drive immune cell recruitment and activation, thereby creating a positive feedback loop that sustains inflammation. FLSs sense the stiffness of the extracellular matrix (ECM) via integrin-FAK-actin remodeling, and activate the YAP/TAZ mechanotransduction pathway [65]. Upon nuclear translocation, YAP/TAZ cooperates with TEAD and AP-1 to directly regulate the expression of ECM components (such as tenascin-C and periostin) and pro-inflammatory factors (such as Cyr61 and CTGF), forming a positive feedback loop. This pathway further promotes Th17 differentiation through RORγt upregulation and stimulates the production of inflammatory cytokines such as IL-6, ultimately driving synovial hyperplasia and bone erosion [66]. Thus, the stiffness of the biomaterials can be designed, and the YAP/TAZ pathway can be targeted to inhibit Th17-mediated inflammation or promote Treg-mediated immune tolerance.

Stiffness has a significant effect on the activation, differentiation, and metabolism of T cells. Studies have shown that T cells can sense chemical signals as well as the elastic and topological structure of their environment. Collectively, these physical signals regulate their immune function. For example, through their adjustable bending modulus (440–20 kPa) and nano-morphology, zinc oxide (ZnO) nanowire arrays regulate the cell contraction force driven by actin polymerization and the mechanical affinity of the “catch-bonds” of the TCR-CD3 complex, affecting the nuclear translocation of NF-κB and regulating IFN-γ secretion, CD107a degranulation, and cytotoxic functions of T cells [67]. The induction rate of Tregs is positively correlated with the stiffness of PA-gels, that is, the harder the substrate, the higher the proportion of induced FOXP3+ Treg cells. Stiffness regulates T cell metabolism through mechanosensing of actomyosin contractility. Hard substrates (140 kPa) directly promote Treg induction by enhancing oxidative phosphorylation (OXPHOS), whereas OXPHOS is insufficient on soft substrates (7.5 kPa), requiring pharmacological activation of AMPK to promote OXPHOS and induce Tregs, although this cannot fully replicate the efficacy of stiff substrates [68]. This indicates that stiffness may be more effective in regulating T cell metabolism than chemical regulatory factors.

In addition, HA, GMHA/MAHA hydrogels, and composite alginate-collagen or alginate-gelatin hydrogels, among other biomaterials, require stiffness adjustment to 5–20 kPa to match the RA synovium. These materials can be used to construct three-dimensional (3D) models that mimic the immune microenvironment of RA synovium and to study the migration and immune regulation behavior of FLSs in different mechanical environments. These models provide an important platform for understanding how mechanical properties indirectly regulate the immune response by affecting the function of the scaffold cells.

In summary, biomaterial stiffness modulates the polarization state, metabolic activity, and phenotypic plasticity of macrophages and T cells through integrin-mediated mechanotransduction, the YAP/TAZ handling pathway, and AMPK-dependent metabolic regulation. This mechanical signaling exacerbates synovial hyperplasia and joint destruction in RA through a positive feedback inflammatory loop involving FLSs and immune cells. However, the threshold effects and mechanisms through which stiffness regulates immune cells, as well as the impact of dynamic stiffness changes in vivo, remain to be elucidated. Future studies could use hydrogels with reversibly tunable stiffness to test whether M1/M2 polarization is reversibly regulated by stiffness cycling. Whether specific knockout of YAP/TAZ in FLSs disrupts the Th17 positive feedback loop requires further investigation. Stiffness-gradient hydrogels could be used to map T-cell subset differentiation-stiffness response curves to determine whether an immune-tolerant stiffness range exists. Furthermore, applying cyclic mechanical loading to hydrogels with a stiffness of 5–20 kPa to mimic the dynamic mechanical microenvironment of the RA synovium would provide a theoretical basis for developing immune-regulatory biomaterials based on stiffness-adaptive design principles.

## 4 Influence of biomaterial morphological structure on immunomodulation

**Fig. 5:**
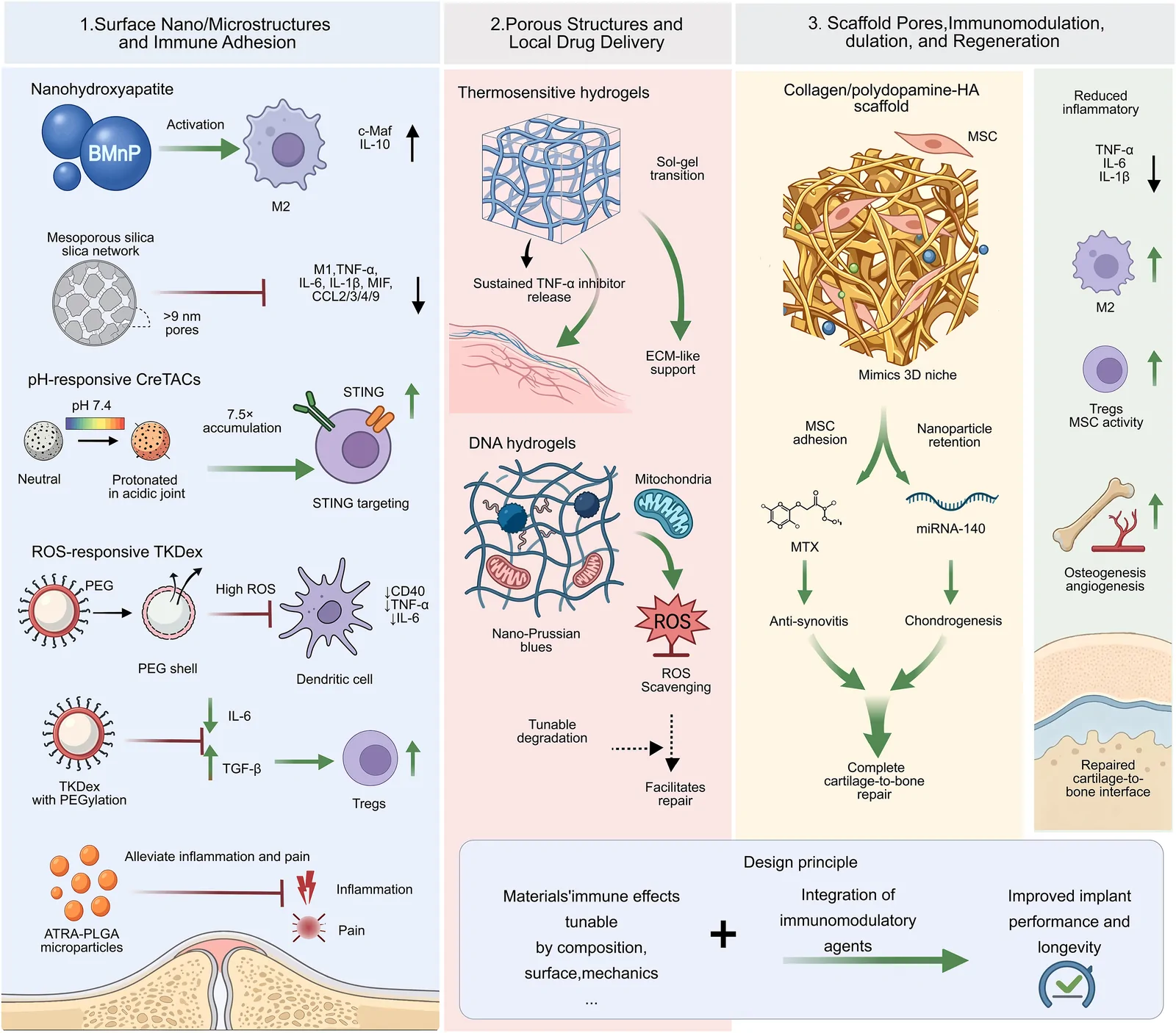
The mechanism for regulating the morphology and structure of biomaterials. pH-responsive CreTACs accumulate 7.5× in acid joints and target STING. Larger hydroxyapatite particles push macrophages to M2 and help bone/blood vessel formation. Mesoporous silica (>9 nm pores) decreases adhesion and cuts inflammatory cytokines. ROS-responsive TKDex sheds PEG, reduces DC CD40 and TNF-α/IL-6; PEGylation decreases IL-6 and increases Tregs. ATRA-PLGA microparticles reduce pain. Hydrogels with 1–100 μm pores slowly release TNF-α inhibitors; DNA hydrogels carry Prussian blue nanoenzymes and mitochondria to clear ROS and help repair. Scaffolds with >100 μm pores hold MSCs and nanoparticles; MTX (fast) and miRNA-140 (slow) together repair cartilage and bone. Therefore, adjusting structure can control immune responses and improve implants.

In the treatment of RA, the morphological properties of the surfaces of biomaterials, such as nanostructures/microstructures, pore structures, and 3D scaffolds, determine their immunomodulatory function. They reshape the inflammatory microenvironment of the joint by regulating the adhesion, migration, polarization, and spatial distribution of immune cells [69] (Fig. 5).

### 4.1 Regulation of immune cell adhesion and polarization by surface nanostructures (1–100 nm)/Microstructures (1–100 μm)

Surface properties at the nanoscale play a crucial role in regulating the behavior of immune cells. Biomedical materials have evolved from passive drug carriers to active immunotherapy agents [69]. These structures function through two pathways: responding to dynamic chemical signals from the RA microenvironment and directly altering the geometric contact between immune cells and the material interface.

The nanostructures precisely target and regulate the expression of stimulator of interferon genes (STING) protein in immune cells by sensing the acidic pH microenvironment of arthritis. Based on this, the charge reversal protein hydrolysis targeting chimeric bodies (CreTACs) platform has been developed. This platform uses the pH-programming property to be electrically neutral in the pH 7.4 blood circulation to reduce systemic toxicity. In an acidic joint microenvironment, it protonates and accumulates in the joint by a factor of 7.5. In addition, it enhances the intracellular retention effect and the affinity for intracellular targets, thereby achieving intelligent and efficient targeted therapy [70]. Similarly, a ROS-responsive nanostructure, TKDex, constructed with a thioketal (TK) linker [71], undergoes PEG shedding in the high-ROS microenvironment of joints with RA. In vitro, it downregulates CD40 expression on DCs and suppresses TNF-α and IL-6 secretion, thereby promoting anti-inflammatory effects. Activated immune cells efficiently internalize the nanostructure after ROS-triggered PEG removal, enabling active targeting of the microenvironment and antigen-specific immune modulation. Furthermore, in an arthritis mouse model, PEG modification prolongs circulation time and enhances joint accumulation, indirectly reducing IL-6 levels, increasing TGF-β, and inducing Treg generation [41]. This type of microenvironmental response design shifts immune regulation from systemic administration to local and on-demand activation.

The nanostructure of the material and the particle size significantly affect the polarization of macrophages. Nanohydroxyapatite (BMnP) particles are more capable of inducing M2 polarization compared to micrometer-sized particles. They enhance IL-10 secretion by activating cMaf and promote osteogenesis and angiogenesis [72]. Simultaneously, mesoporous silica with pore sizes of >9 nm can form a fine fiber network, reducing the macrophage contact area. By inhibiting cell adhesion and scaffold assembly, they reduce M1 polarization and the expression of pro-inflammatory factors TNF-α, IL-6, IL-1β, and MIF, as well as chemokines CCL2, CCL3, CCL4, and CCL9, providing a solution for regulating coagulation and immune regenerative materials [73]. Notably, nano-topology can directly regulate immune cells without relying on drug release.

Furthermore, micro-scale structures also possess the potential for immune regulation. Intra-articular injection of all-trans retinoic acid (ATRA)-loaded poly(lactic-co-glycolic acid) (PLGA) microparticles (ATRA-PLGA MP) alleviates inflammation and reduces mechanical pain hypersensitivity in a mouse model of RA [74].

In conclusion, nano/micro surface structures enable microenvironment-targeted immune intervention through pH/ROS responsiveness and directly modulate immune cell adhesion and polarization through geometric parameters such as size, pore diameter, and morphology, thus reflecting a shift from passive carriers to active regulators. However, key issues remain. The causal link between chemical changes and immune effects is largely inferred; for instance, whether PEG detachment from TKDex directly drives DC tolerance and Treg induction lacks nonresponsive controls and real-time evidence. Moreover, systematic quantitative relationships between geometric parameters and immune polarization have yet to be established. Future studies should construct libraries of particles with continuously tunable size and pore diameter, map dose response curves linking structural parameters to immune polarization in RA models, and verify causality through YAP/TAZ blockade. These efforts would provide a basis for the design of immune regulatory materials based on geometric topology.

### 4.2 Pore structures (1–100 μm) and their regulation of inflammatory microenvironment and drug delivery

Based on their functional properties corresponding to the pore size, this section and Section 4.3 separately discuss small pore structures designed primarily for drug-controlled release and immune modulation and large pore structures designed primarily for cell scaffolding and tissue regeneration. In practical design, these two types often coexist and work synergistically to achieve integrated therapy throughout the treatment process.

The porous structure, through its 3D interconnected network, significantly influences the local retention and release dynamics of the drug, enabling continuous regulation of the inflammatory microenvironment. Injectable thermosensitive hydrogels are representative of this area. These gels can undergo reversible sol-gel transformation at physiological temperature, forming a structure with a porous network and achieving minimally invasive injection and long-term sustained release of drugs in the joint cavity [29]. For instance, thermosensitive hydrogels based on PLGA-PEG-PLGA triblock copolymers, pluronic micelles, or hyaluronic acid are used for the continuous intra-articular delivery of TNF-α inhibitors to maximize local efficacy and minimize systemic side effects. The biocompatibility, degradability, and tunable mechanical properties of these hydrogels can simulate ECM to support joint repair [29]. Polymer-modified DNA hydrogels use their porous structure as a drug reservoir to encapsulate Prussian blue nanoenzymes and active mitochondria. They collaboratively eliminate exogenous and endogenous ROS, remodel the RA inflammatory microenvironment, and support osteochondral repair through a tunable degradation rate [75].

Although pore structures in the 1–100 μm range hold promise in drug delivery and microenvironment remodeling, existing studies have largely relied on material characterization and single-parameter assessments, resulting in the quantitative relationships between structural parameters and macrophage polarization, Th17/Treg balance, and FLS activation being poorly defined. Furthermore, small-pore designs alone are insufficient to interrupt the multicellular inflammatory positive feedback in RA, necessitating integrated macroporous scaffold designs, as detailed in Section 4.3.

### 4.3 Synergistic effects of scaffold pore structures of >100 μm in immunomodulation and tissue regeneration

The scaffold structure shows unique advantages in regulating the spatial distribution of immune cells and promoting tissue regeneration by simulating the complex spatial microenvironment of natural tissues. The scaffold is constructed from collagen and polydopamine modified hyaluronic acid. It promotes attachment of mesenchymal stem cells and prolongs the retention time of drug-loaded nanoparticles in the joint, providing a foundation for the establishment of a regenerative microenvironment. This scaffold integrates inflammatory-responsive methotrexate (MTX) nanoparticles and a miRNA-140 delivery system that promotes cartilage formation, thereby achieving a two-stage treatment. MTX is rapidly released under inflammatory conditions to inhibit synovitis (the immunomodulatory stage), after which the scaffold continuously releases miRNA-140 to provide structural support and biological signals for cartilage regeneration. A complete restoration of bone-cartilage structure from transparent cartilage to subchondral bone repair was ultimately achieved in the RA animal model [76]. This marks a shift in the treatment approach from symptom management to true disease-modifying and functional tissue regeneration [76].

The immune response triggered by engineered materials can be regulated by their composition, surface properties, or mechanical properties. This is the core strategy for designing the next generation of implants [77]. Integration of immunomodulatory molecules or cell therapies into scaffold biomaterials can more precisely control the host response, thereby improving the functionality and lifespan of the implant [77].

However, current scaffolds still have limitations: morphology, size, and pore diameter are studied in isolation, necessitating standardized comparisons. Research is macrophage-centered and largely ignores FLSs (key effector in RA). Notably, high porosity enhances infiltration but impairs joint-loading strength, and surface roughness effects are material-dependent. Micron roughness on titanium/PTFE modulates macrophages and cytokines, whereas polyurethane is chemistry-driven, with roughness having little effect [78]. Although the delivery of the MTX + miRNA-140 stent achieved a two-stage treatment, its clinical application requires verification of the long-term safety of the PLGA by-products, individualized differences in ROS/pH response thresholds, and whether the stent can uniformly adhere to the diseased tissue.

In summary, microporous structures focus on drug delivery and immune microenvironment remodeling, whereas macroporous scaffolds prioritize tissue regeneration and host integration. Although complementary within the porous system, both remain confined to single-parameter, single-cell studies. Future designs should synergistically optimize nanotopography, pore architecture, and mechanical support, leveraging 3D printing for personalized multi-scale customization. This would shift materials from passive physical structures toward active immunomodulation, thus providing a theoretical basis for next-generation immunomodulatory regenerative scaffolds.

## 5 Regulation of RA-related immune cells by the physicochemical properties of biomaterials

The composition and interactions of immune cells in the microenvironment of RA play a pivotal role in pathogenesis. The principal immune cells comprise macrophages, DCs, T cells, neutrophils, B cells, and NK cells, which play a crucial role in the pathogenesis of RA [[79], [80], [81]]. Table 1, Table 2, Table 3, Table 4 present the types of biological materials, the immune cells they act upon, their physical and chemical properties, the immune regulation mechanisms, and the key signaling pathways.

**Table 1:** Regulation of macrophage polarization mediated by biomaterials.

Ref.	Biomaterial Type	Physicochemical Properties	Targeted Immune Cells	Active Immunomodulatory Mechanism	Signaling Pathway	Key Inflammatory Factors (Upregulated)	Key Inflammatory Factors (Downregulated)	Study Model/Key Results
[82]	P(VDF-TrFE) coating	Positive charge	Bone marrow-derived macrophages	High potential promotes M2 polarization	Integrin and K+ channel	M1: iNOS, CD80, TNF-α, IL-1β; M2: CD206, IL-10, IL-4	Not mentioned	In vitro: BMDMs, high potential favors M2
[83]	Silk Fibroin Film	Surface Roughness	Macrophages	High roughness → M2; low roughness → M1	Integrin αv-NF-κB	TNF-α, IL-6 (low roughness)	IL-10, TGF-β (high roughness)	In vitro: RAW264.7; In vivo: mouse subcutaneous implant. Rough SF mitigates early inflammation
[84]	Gelatin nanofibrous matrix	Nanofibrous architecture	Macrophages (RAW264.7)	Attenuates M1, upregulates cell-matrix interaction, maintains non-polarized state	ECM-receptor interaction (up); NF-κB & NOD-like (down)	Not mentioned	iNOS, Tnfaip3, Il6, Cxcl2, Marco, Il1b, Il1a, Il12b	In vitro: RAW264.7; transcriptome shows up-regulated ECM-receptor, down-regulated pro-inflammatory pathways
[85]	Nanosilicate-reinforced hydrogel + halimine	Nanosilicate carrier, sustained release	Macrophages	Induces M2, creates anti-inflammatory microenvironment	Not mentioned	Not mentioned	Pro-inflammatory factors (unspecified)	Promotes osteogenesis
[86]	Hyaluronic acid-modified PtPdCo-CQ nanocatalyst (HPPCQ)	Mesoporous, ∼140 nm, SOD/CAT/POD, −38.83 mV, CQ loading/release	M1 macrophages (HA-CD44), protects M2	Scavenges ROS, promotes M1→M2; releases CQ, inhibits M2 autophagy-dependent ferroptosis	Nrf2/HO-1; inhibits JAK-STAT, NF-κB	TNF-α, IL-1β, IL-6	Not mentioned	CIA model: reduced clinical scores, synovitis, cartilage destruction. Decreased serum TNF-α, IL-1β
[87]	Titanium	Surface roughness, hydrophilicity (smooth, rough, rough-hydrophilic)	Macrophages	Surface regulates Wnt ligand secretion, modulates M1/M2	Wnt signaling	IL-1β, IL-12, TNF-α, CXCL-10 (Wnt3a, Wnt5a)	IL-4, IL-10 (Wnt5b, Wnt11)	In vitro: macrophage Wnt expression by surface; in vivo: macrophages main Wnt source, loss impairs MSC/T-cell recruitment
[88]	Fe/Zn-doped silicon apatite (Fe-CPS/Zn-CPS)	Slow Fe^2+^ release, rapid Zn^2+^ release	Macrophages	Promotes M2, inhibits M1	Fe-CPS: PI3K-Akt, JAK-STAT, Hippo; Zn-CPS: Rap1, complement, NF-κB	Not mentioned	Not mentioned	In vitro: qRT-PCR, RNA-seq, flow, etc.; in vivo: rat calvarial defect. Fe-CPS best bone at day 14; Zn-CPS faster M2 and bone formation
[89]	PEGDA hydrogel	SOCS1-KIR peptide	Macrophages (Raw264.7, human monocytes)	SOCS1-KIR inhibits JAK phosphorylation, reduces M1, promotes M2	JAK/STAT	None	iNOS, TNFα, CD86, CD38	2D/3D cultures; peptide reduces M1 markers, upregulates CD206 in human macrophages
[90]	PPFU grafted with PLL/PDL	Grafted with polylysine, enhanced wettability	Macrophages	Promotes M2, restricts M1 via CD44/integrin adhesion	PI3K/Akt1/mTOR	IL-10, TGF-β	TNF-α, IL-1β	In vitro: BMDMs M2; inhibits LPS/IFN-γ-induced M1. In vivo: thinner fibrous capsule
[91]	Biomimetic carrier (DAMRT)	Macrophage membrane-encapsulated mitochondria + AUTAC4 + DSPE-PEG-FA	M1 macrophages	Transfers healthy mitochondria; clears dysfunctional mitochondria via AUTAC4	Not specified	Not specified	Not specified	RA model: restores mitochondrial homeostasis, regulates macrophage polarization
[92]	Mitochondria-targeting biomaterials (concept)	Not mentioned	Macrophages	Mitochondria-targeted immunomodulation	Not mentioned	Not mentioned	Not mentioned	Not mentioned
[93]	Selenium-doped mesoporous bioactive glass (Se-MBG)	Mesoporous structure, can be doped with selenium ions	Macrophages, T cells	Promotes M2 polarization, restores M1/M2 balance; promotes Treg differentiation, inhibits Th17 differentiation, corrects Treg/Th17 imbalance	Not mentioned	IL-17A27	IL-10, TGF-β27	RA model; decreased M1 macrophages, increased M2 macrophages; increased Foxp3+ Treg density, decreased IL-17A + Th17 infiltration in synovium
[94]	CaO_2_/CeO_2_@ZIF-8-ALN-AOA nanocomposite	Acid-responsive, bone-targeted, nanostructured	Macrophages (inflamed joint)	Disintegrates in acid → neutralizes H^+^, produces O_2_, enhances antioxidant → M1→M2	Not mentioned	Not mentioned	Not mentioned	Not mentioned
[95]	MXenzyme-enhanced hydrogel	Multi-antioxidant enzyme-like (SOD, CAT, GPx); scavenges ROS/RNS, generates O_2_	Macrophages (M1→M2); BMSCs	Scavenges ROS/RNS, alleviates hypoxia, induces M1→M2, protects BMSCs	Not mentioned	Not mentioned	Not mentioned	Rabbit RA: reduced synovitis, promoted bone remodeling, improved BV/TV, Tb.N, Tb.Th, decreased Tb.Sp

**Table 2:** Regulation of DCs immune tolerance mediated by biomaterials.

Ref.	Biomaterial Type	Physicochemical Properties	Targeted Immune Cells	Active Immunomodulatory Mechanism	Signaling Pathway	Key Inflammatory Factors (Upregulated)	Key Inflammatory Factors (Downregulated)	Study Model/Key Results
[38]	Polyethyleneimine-modified nanoparticles	Positive charge	Immune cells (indirectly via cfDNA scavenging)	Scavenging cfDNA, inhibiting immune cascades	TLR9 (indirectly inhibited by removing ligand)	TNF-α, IL-6, IL-1β (elevated in model)	TNF-α, IL-6, IL-1β (reduced in cNP2 group)	CIA rat model; cNP2 inhibited swelling, infiltration, pannus, bone/cartilage damage
[96]	Modified zinc peroxide nanoparticles (ZnCM NPs)	pH-responsive release of Zn^2+^ and O_2_; surface modification with mannose for DC targeting	DCs	Induces tolerogenic DCs (tDCs) by inactivating OTUB1 deubiquitination, promoting CCL5 degradation via NF-κB signaling, and suppressing immunogenic DC (igDC) activation	NF-κB signaling pathway	None (pro-inflammatory factors are suppressed)	IL-1α, IL-1β, CCL5, IL-6, IL-12α, IL-12β, TNF-α	CIA mouse model: significantly alleviated ankle swelling, improved walking posture, and inhibited inflammation in the ankle and spleen
[97]	CuS	nanoparticles	Activated T cells, macrophages, DCs	Induces cuproptosis of activated T cells → fragments engulfed by macrophages → abundant TGF-β production; TGF-β synergizes with Rapa to convert iDCs into tDCs; tDCs present CitP to naive T cells → promote Treg differentiation; Tregs produce more TGF-β, establishing a self-sustaining immune tolerance cycle	Not mentioned	TGF-β	Not mentioned	CIA mouse model; Treating RA by inducing T cell cuproptosis

**Table 3:** Regulation of T cell subset functions mediated by biomaterials.

Ref.	Biomaterial Type	Physicochemical Properties	Targeted Immune Cells	Active Immunomodulatory Mechanism	Signaling Pathway	Key Inflammatory Factors (Upregulated)	Key Inflammatory Factors (Downregulated)	Study Model/Key Results
[98]	BMSC-exosome	Not mentioned	CD4^+^ T cells	Delivering JKAP to restore Th17/Treg balance	AKT, ERK	IL-6, IL-8, CCL2	IL-10	CIA model: JKAP-overexpression exosomes alleviated synovial hyperplasia and inflammation
[99]	MSC-derived exosomes (Exo-RA)	Size: ∼112.4 nm; Number: 5.94 × 10^10^ per 10^7^ iMSCs	Macrophages, T cells (Th1, Th17, Th2, Treg), B cells	Yes. Actively reshapes immune microenvironment via TGF-β1 and PGE2, inducing Th2 and M2 polarization.	TGF-β/NF-κB; PI3K-AKT; cAMP	IL-437; TGF-β1; IL-10	TNF-α; IL-1β; IL-6; IL-17; KC; IL-12p70	Model: CIA mice. Key Results: Reduced arthritis scores & paw swelling; Lowered serum TNF-α, KC, IL-12p70; Increased serum IL-4; Increased M2 macrophages; Suppressed T cell proliferation.
[100]	PIC polymer scaffold	Semi-flexible structure; functionalizable with multiple molecules via click chemistry	ACPA-expressing autoreactive B cells	Co-engagement of BCR and CD22 to actively downregulate B-cell activation	BCR-CD22 co-signaling: inhibition of Syk phosphorylation	Not directly measured	Not directly measured	Model: ACPA-BCR Ramos B cells and RA patient PBMCs. Results: 1. Successful detection of ACPA-B cells. 2. CCP/CD22L PICs significantly inhibited Syk phosphorylation.

**Table 4:** Regulation of neutrophils, B cells, and NK cells immune responses mediated by biomaterials.

Ref.	Biomaterial Type	Physicochemical Properties	Targeted Immune Cells	Active Immunomodulatory Mechanism	Signaling Pathway	Key Inflammatory Factors (Upregulated)	Key Inflammatory Factors (Downregulated)	Study Model/Key Results
[101]	SA-modified liposomes (DP-SALs)	Smaller size (DP-SAL-S) has stronger targeting	Neutrophils (PBNs) and macrophages	1. SA targets PBNs. 2. Released DP induces macrophage cytotoxicity.	Not mentioned	IL-6, TNF-α	IL-10, TGF-β	1. DP-SAL-S showed highest accumulation in arthritic joints. 2. Significantly inhibited arthritis, DP-SAL-S most effective.
[100]	PIC polymer scaffold	Semi-flexible structure; functionalizable with multiple molecules via click chemistry	ACPA-expressing autoreactive B cells	Co-engagement of BCR and CD22 to actively downregulate B-cell activation	BCR-CD22 co-signaling: inhibition of Syk phosphorylation	Not directly measured	Not directly measured	Model: ACPA-BCR Ramos B cells and RA patient PBMCs. Results: 1. Successful detection of ACPA-B cells. 2. CCP/CD22L PICs significantly inhibited Syk phosphorylation.
[102]	AuNPs, Hypoxis-AuNPs, Hy-AuNPs	Spherical, avg. 26 ± 2 nm	Macrophages (THP1), NK cells (NK92)	Not mentioned	Not mentioned	Not mentioned	Macrophages: IL-1β, TNF-α; NK cells: IFN-γ (only Hy-AuNPs)	In vitro. All treatments reduced IL-1β and TNF-α in LPS-stimulated macrophages; only Hy-AuNPs reduced IFN-γ in NK92 cells.

### 5.1 Regulation of Macrophage polarization by biomaterials in RA

Macrophages are differentiated into pro-inflammatory M1 and anti-inflammatory M2 forms [[103], [104], [105]]. Balancing these two types is crucial for controlling RA. M1 releases pro-inflammatory factors such as TNF-α, IL-1β, and IL-6, which exacerbate joint inflammation and bone destruction. M2 secretes anti-inflammatory factors, promoting repair and alleviating inflammation [106,107]. Physicochemical properties of biomaterials, such as hydrophilicity, charge, and nanostructure, can modulate their polarization direction by affecting receptors and signaling pathways (Table 1).

Cell phenotype and functional regulation. Surface properties of materials, including charge, roughness, and nanostructure, directly affect macrophage morphology, adhesion, and membrane curvature. For instance, positively charged poly(vinylidene fluoride-trifluoroethylene) [P(VDF-TrFE)] coatings can enhance the polarization of bone marrow-derived macrophages toward the anti-inflammatory M2 type through integrin signaling pathways [82]. The surface of SF films with high roughness has been shown to induce positive curvature in the plasma membrane of macrophages, thereby promoting the endocytosis of integrin αv and resulting in its decreased expression. The endocytic degradation of integrin αv inhibits the integrin- NF-κB signaling pathway, as evidenced by reduced levels of p-FAK and p-NF-κB, along with decreased secretion of pro-inflammatory factors such as TNF-α and IL-6. This inhibition ultimately drives macrophage polarization towards the M2 anti-inflammatory phenotype, thereby alleviating the inflammatory response [83]. Nanofiber-based biomimetic ECM scaffolds guide macrophages toward an anti-inflammatory phenotype by regulating gene expression profiles [84]. Biomaterials function as inert scaffolds. In addition, their physical and chemical properties actively make macrophages change their functions through the combined effects of membrane curvature, integrins, and inflammatory signals.

The regulatory effect of the material is ultimately reflected in the changes of key inflammatory factor levels. Nanosilicate loaded with halimine enhances the hydrogel, which can effectively induce macrophages to polarize towards M2, creating an anti-inflammatory microenvironment conducive to tissue repair, thereby inhibiting the release of pro-inflammatory factors and promoting osteogenesis [85]. Layered double hydroxides, nanocatalysts, and particles, amog other specific nanomaterials can target M1 macrophages, regulate their polarization state, reduce the release of inflammatory factors, and thereby alleviate the inflammatory response in RA [86,108,109]. These studies integrate immune regulation and tissue repair onto the same delivery platform.

Molecular signaling pathway regulatory mechanisms. The surface properties of biomaterials can regulate the Wnt signaling pathway, impacting macrophage polarization [87]. Doping calcium silicate ceramics with different metal ions activates distinct immune regulatory mechanisms. Iron ions activate the PI3K-Akt and JAK-STAT signaling pathways to promote M2 polarization [88], whereas zinc ions primarily inhibit M1 polarization by upregulating pathways such as Rap1 [88]. The sustained release of SOCS1 peptide segments in hydrogels can effectively inhibit the JAK/STAT signaling pathway, thereby reducing the activation of pro-inflammatory M1 [89]. Polyurethane membranes modified with peptides can promote M2 polarization by activating the PI3K/Akt1/mTOR signaling axis [90]. In summary, pathways such as Wnt, NF-κB, JAK/STAT, MAPK, and PI3K/Akt are all involved in the regulation of the balance between M1 and M2 polarization.

Macrophage polarization is influenced by the physical and chemical properties of the biomaterials and critically depends on their metabolic state and mitochondrial function. M1 relies primarily on glycolysis, whereas M2 relies on oxidative phosphorylation. Mitochondrial biomimetic vectors precisely target the activated M1 macrophages in the joints. They achieve mitochondrial renewal and restoration of cellular homeostasis by delivering healthy mitochondria and eliminating dysfunctional mitochondria [91]. Targeting mitochondrial-based biological materials for immunomodulation has become a new direction [92]. For example, selenium-doped mesoporous biomaterials can clear ROS, improve mitochondrial function, promote M2 polarization, and thereby enhance bone regeneration [93,110]. Targeting mitochondria for immune regulation is emerging as a new research direction.

In recent years, intelligent biomaterials have been designed to further enable responsive regulation of the lesion microenvironment. For instance, a bone-targeted acid-responsive nanocomposite (CaO_2_/CeO_2_@ZIF-8-ALN-AOA) can disintegrate under acidic conditions of inflamed joints. By neutralizing excess H^+^, generating oxygen and enhancing antioxidant activity, it effectively promotes the polarization of macrophages from M1 to M2 [94]. The hydrogel enhanced by MXene nanozymes can remove excessive ROS/active nitrogen species (RNS) and synergistically produce oxygen, inducing M2 polarization in the oxidative microenvironment [95]. Microenvironmental cues act as endogenous triggers, enabling on-demand, localized immune regulation.

In summary, the physicochemical properties of biomaterials modulate the expression of surface receptors and signaling pathways of macrophages through various mechanisms, modulate the M1/M2 balance, affect the levels of key inflammatory factors, and inhibit inflammatory processes in RA.

### 5.2 Regulation of DC maturation and tolerance by biomaterials in RA

DCs are APCs. The physicochemical properties of biomaterials affect the maturation of DCs and the expression of co-stimulatory molecules, thereby regulating the efficiency of antigen presentation and immune tolerance. Surface properties such as charge and functional modifications of the material can regulate the activation state and expression of co-stimulating molecules of DCs. Nanoparticles modified with positively charged polyethyleneimine can enhance the uptake and presentation efficiency of antigens by DCs [38]. The materials can be designed to release immunomodulatory factors in response to the characteristics of the inflammatory microenvironment, such as high ROS levels, further enhancing the function of DCs [41] (Table 2).

Induced immunological tolerance DCs (tDCs) are an effective means of suppressing the autoimmune T-cell response in RA [111]. Modified zinc oxide nanoparticles (ZnCM NPs) release Zn^2+^ and O_2_ in an acidic cellular environment. After this, they inactivate the OTUB1 deubiquitinating enzyme and, through the NF-κB signaling pathway, promote the degradation of CCL5. This process inhibits the immunogenicity of DCs; reduces the expression of co-stimulatory molecules CD80, CD86, and MHC II class molecules; and inhibits the mRNA transcription of pro-inflammatory factors IL-1, IL-6, IL-12α, IL-12β, and TNF-α. The induced tDCs can inhibit the activation of downstream CD4^+^ T cells and alleviate the immune response in RA [96].

In addition, antigen-specific immune tolerance is a novel approach to the treatment of RA [112,113]. CuS NPs induce Cuproptosis of activated T cells, whose fragments are engulfed by resident or recruited macrophages, resulting in abundant production of TGF-β. TGF-β acts synergistically with Rapa to induce the iDCs into tDCs. tDCs present CitP to naive T cells, promoting Tregs differentiation. Tregs, in turn, produce more TGF-β, inducing tDCs differentiation, thereby establishing a cycle of immune tolerance [97].

Biomaterials modulate the activation state and antigen presentation function of DCs through methods such as surface charge, response release and signal pathway intervention. Inducing tDCs can also inhibit T cells. Depending on the material-regulated activation mechanism of DCs, specific materials can optimize the antigenic presentation function of DCs by modulating their metabolism and signaling pathways, thereby enhancing the specificity and efficacy of immunotherapy.

### 5.3 Regulation of T cell subset functional balance by biomaterials in RA

Imbalance in the functional activities of T cell subsets represents a central feature of the immune pathology underlying RA [114]. The physicochemical properties of biomaterials can modulate the differentiation and function of these T cell subsets by influencing the metabolic status, cytokine secretion, and transcriptional regulatory networks within the immune microenvironment, with particular emphasis on maintaining the equilibrium between Th17 and Treg cells [98]. Specifically, Th17 cells secrete pro-inflammatory cytokines such as IL-17 that promote inflammatory responses and tissue damage (Table 3).

Upon targeted delivery to joint tissues, the acid-responsive nanocomposite undergoes degradation to release glyoxylic acid (AOA), thereby promoting Treg cell differentiation and restoring Th17/Treg axis balance [94]. In parallel, exosomes derived from BMSCs deliver JKAP protein through the Ak strain thymoma (AKT)/extracellular signal-regulated kinase (ERK) signaling pathway to re-establish Th17/Treg equilibrium, ultimately alleviating RA symptoms [98].

The most prevalent mechanism involves indirect regulation through APCs. For instance, MSC-derived exosomes reprogram macrophages from the M1 to the M2 phenotype [115], thereby indirectly promoting Treg expansion or suppressing Th17 differentiation [99]. In addition, Kristyanto et al. used a multivalent polyisocyanopeptide (PIC) scaffold to deplete pathogenic B cells, which consequently abrogated self-antigen presentation and prevented autoreactive T cell activation [100].

Alterations in fatty acid metabolism, for example, have been associated with a pro-inflammatory phenotype in CD8+T cells in RA patients. Pharmacological inhibition of fatty acid metabolism attenuates this pro-inflammatory activity, indicating that lipid metabolism represents a potential target for modulating T cell function [116]. In parallel, oral administration of ginger-derived extracellular vesicles (GDEVs) enables the delivery of miR-149, which suppresses inflammatory signaling pathways and elicits systemic anti-inflammatory effects, thereby indirectly modulating T cell responses [117].

Biomaterials use multimodal mechanisms to regulate T cell fate: direct targeting of differentiation pathways, indirect orchestration of immune responses through APC cascades, and reprogramming of metabolic states. Collectively, these strategies effectively re-establish the Th17/Treg equilibrium, furnishing a molecular framework for the precise manipulation of T cell function in RA immunotherapy.

### 5.4 Regulation of neutrophil, B cell, and NK cell immune responses by biomaterials in RA

In addition to DCs and T cells, neutrophils, B cells, and NK cells are also involved in immune regulation in RA. Biomedical materials can indirectly modulate these immune cells through targeted delivery, receptor cross-linking, and surface modification, thereby jointly alleviating joint inflammation (Table 4).

Neutrophils massively infiltrate the joints during the early stages of RA, and their degranulation and reactive oxygen species release constitute a major mechanism underlying cartilage and bone destruction. SA mediates the recognition and internalization of small-sized (75 nm) liposomes by PBNs through its specific binding to L-selectin. Although the uptake is relatively low, the liposomes retain the adhesion, chemotaxis, and NETs formation capabilities of the cell, enabling efficient inflammatory targeting and drug delivery. In contrast, large-sized liposomes exhibit high uptake but significantly inhibit these functions, thereby reducing delivery efficiency [101].

The PIC scaffold uses a bi-targeting strategy for antigen-specific immunomodulation of B cells. By co-displaying cyclic citrullinated peptide antigen and CD22 ligand, the scaffold drives spatial co-localization of the B-cell receptor (BCR) with the inhibitory receptor CD22. This architecture enables concurrent BCR activation and active recruitment of inhibitory signaling, wherein CD22 engagement suppresses Syk phosphorylation to antagonize BCR-driven activation, ultimately reducing B-cell effector function [100]. Gold nanoparticles functionalized with surface-encapsulated phytochemicals further modulate innate immune responses by markedly attenuating IFN-γ secretion in NK92 cells, thereby conferring anti-inflammatory properties [102].

Through rational particle size engineering, biological materials reconcile the trade-off between neutrophil function preservation and delivery efficiency. A dual-ligand scaffold enables targeted inhibition of pathogenic B cells, whereas phytochemically functionalized nanoparticles modulate NK cell cytokine secretion. Collectively, these strategies achieve indirect yet precise regulation across diverse innate and adaptive immune compartments, thereby expanding the therapeutic target space for RA immunotherapy.

In Sections 5.1–5.4, it is discussed that biomaterial regulation of the RA immune microenvironment now spans macrophages, DCs, T cells, neutrophils, B cells, and NK cells, covering innate and adaptive immunity. Strategies include physicochemical cues, molecular delivery, metabolic reprogramming, and smart responsive design, with mechanistic insights advancing from phenotype descriptions to signaling pathways and metabolic phenotypic coupling. However, certain limitations remain: charge, stiffness, and pore size are often studied jointly, hindering independent analysis of their immune effects; mechanistic validation relies mostly on in vitro single-cell systems, lacking multicellular interactions among FLSs, T cells, and osteoclasts within the joint, as well as extended in vivo validation; and standardized assessment of response thresholds, targeting selectivity, and safety over time is absent for metabolic regulation and smart materials. Future work should independently tune these parameters on a unified platform, combined with multicellular coculture, RA animal models, and single cell sequencing, to quantify their effects on immune cell function and inflammation and to identify optimal parameter combinations for guiding immunomodulatory material design.

### 5.5 Regulation of the FLS–immune cell vicious cycle by biomaterials in RA

**Fig. 6:**
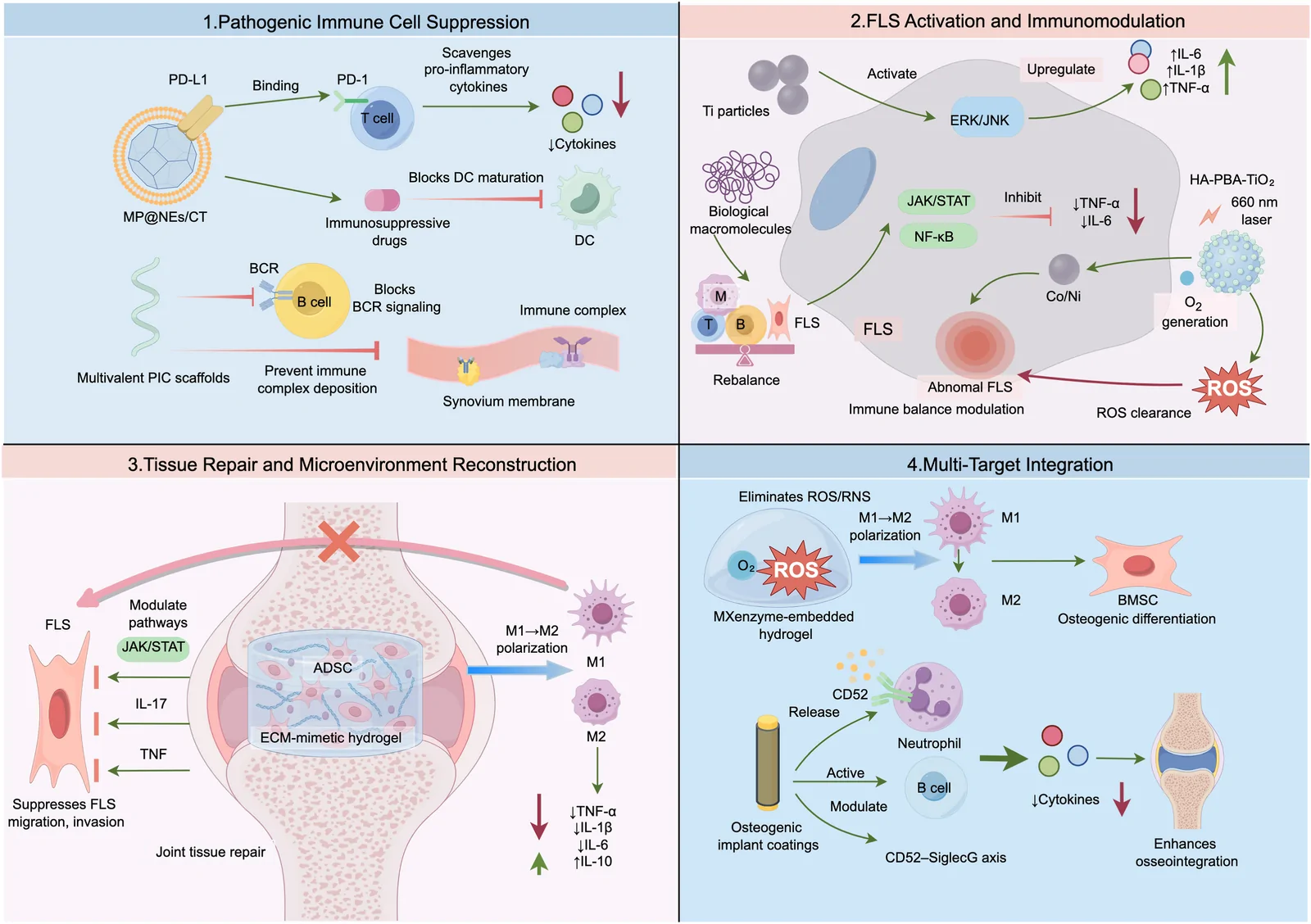
Relationship between FLSs and immune regulation. Nanoparticles block T cells, DCs, and BCR signaling to reduce inflammation. Ti particles activate FLSs; macromolecules and TiO_2_ nanocomposites inhibit FLSs through JAK/STAT/NF-κB, ROS clearance, and photothermal ablation. Hydrogels with ADSCs or MXenzyme promote M1 to M2 polarization, osteogenesis, and tissue repair; implant coatings improve osseointegration through CD52. The picture was drawn by figdraw.com.

In the RA synovial microenvironment, FLSs and infiltrating immune cells establish a bidirectional, self-amplifying feedback loop that propels progressive joint destruction [118]. Biomaterial-based interventions dismantle this pathogenic cycle through four interconnected mechanistic modalities [119,120] (Fig. 6).

One aspect is to inhibit the generation and activation of pathogenic immune cells in the upper part. Zhong et al. engineered macrophage membrane-coated nanomedicines (MP@NEs/CT) that leverage PD-L1-PD-1 interactions to achieve targeted accumulation at T cells, while concurrently scavenging pro-inflammatory cytokines. The therapeutic payload further arrests DC maturation, thereby synergistically inducing antigen-specific immune tolerance [121]. Complementing this strategy, multivalent PIC scaffolds directly engage the BCR signaling cascade to block self-antigen presentation and immune complex deposition within synovial tissue [100].

The second approach is to directly regulate the functional and phenotypic transformation of the FLS effect. Titanium particles activate ERK and JNK MAPK pathways in FLSs, markedly upregulating IL-6, IL-1β, and TNF-α [122]. Conversely, biological macromolecules rebalance the T cell–macrophage–B cell–FLS axis by targeting JAK/STAT and NF-κB signaling, modulating TNF-α and IL-6 production, and leveraging the RA-specific microenvironmental hallmarks, including ROS, low pH, and high MMP activity, for precision immunotherapy [123]. The HA-PBA-TiO_2_ nanocomposite exemplifies multimodal FLS targeting: under 660 nm irradiation, it photocatalytically generates O_2_ to ameliorate hypoxia and oxidative stress, whereas Co/Ni doping enables photothermal conversion for selective FLS ablation, achieving concurrent immunomodulation and therapeutic efficacy [124].

The third is tissue repair and microenvironment reconstruction, which disrupts the positive feedback between FLSs and immune cells. An ECM-mimetic hydrogel encapsulating adipose-derived stem cells (ADSCs) orchestrates multi-pathway regulation (JAK/STAT, IL-17, and TNF-α) to suppress FLS migration, invasion, and pro-inflammatory secretion. Simultaneously, it reprograms macrophage polarization from the M1 to the M2 phenotype, reducing TNF-α, IL-1β, and IL-6, while elevating IL-10. This dual action severs the FLS–M1 macrophage positive feedback loop, culminating in joint tissue repair and protection [125].

The fourth aspect is the multi-target regulation of biological materials. Multi-target integration: from single nodes to network pharmacology. Emerging paradigms favor single material, multi-node interventions. MXenzyme-embedded hydrogels eliminate ROS/RNS, synergistically generate O_2_, drive M1 to M2 macrophage repolarization, and promote BMSC osteogenic differentiation, collectively mitigating joint damage [95]. Similarly, osteogenic implant coatings induce neutrophil CD52 release, while modulating the CD52-SiglecG axis, B cell activation, and inflammatory mediator secretion to enhance osseointegration [126].

By converging on BCR signaling, JAK/STAT, IL-17, TNF-α, and CD52 pathways, biomaterials operate at multiple network nodes within the FLS–immune cell interactome, transforming the pathogenic vicious cycle into a homeostatic, reparative process. However, the contribution of each target in multi-target materials remains unclear, making it difficult to distinguish synergistic effects from single-target dominance. Excessive immunosuppression may also predispose to infection or impaired repair. In addition, photothermal killing of FLSs can release damage-associated molecular patterns and activate residual immune cells, a side effect that has not been systematically evaluated. This multi-target paradigm reverses pathological progression as well as establishes a molecular framework for rational RA immunomaterial design, thus bridging mechanistic insight with translational therapeutic engineering.

## 6 RA immunomodulatory strategies and applications based on biomaterials

In the precision treatment of RA, the physicochemical properties of biomaterials, such as degradability, surface energy, mechanical properties, charge, and topography, are core design parameters that determine drug-release kinetics, direct immune cell behavior, and reshape the joint microenvironment.

### 6.1 Controlled degradation and stimuli-responsive properties of drug delivery systems

By rationally engineering the chemical structure of biomaterials, on-demand drug release can be achieved. The core of degradation-controlled release lies in the intrinsic physicochemical properties of the material. Through passive, continuous degradation, drugs are gradually released at a pre-programmed rate, rendering it suitable for long-term treatment without repeated administration [127]. For instance, by adjusting the monomer ratio (LA:GA) and molecular weight of poly(lactic-co-glycolic acid) (PLGA), the hydrolysis rate can be programmed, allowing drug release from the carrier to be tuned from 1 to 6 months [128].

Stimuli-responsive release is triggered by external or internal environmental signals—such as pH, temperature, and enzyme activity—to act as switches for active drug release [129]. This enables precise release at target sites, such as acidic inflammatory tissues, thereby enhancing targeting and reducing side effects. Furthermore, external stimulus-responsive systems provide a novel avenue for on-demand, precise drug release. For example, upon electrical stimulation, melanin nanoparticles (FMNPs) exhibit markedly enhanced drug release [130].

Depending on the delivery target, drug delivery systems can be broadly classified into two categories: nanoparticles and injectable microspheres. Biomimetic membrane–coated nanocarriers can prolong circulation time and actively target inflamed joints [131,132], whereas the injectable microsphere system can achieve long-term sustained release of drugs and improve patient compliance [133,134]. Carrier particle size and surface charge are key parameters that regulate tissue penetration, cellular uptake, and in vivo stability [135,136].

The biomimetic membrane carrier can achieve systemic administration, targeted delivery to joints and endocytosis, but the drug loading capacity is limited. Its safety needs to be verified and it relies on passive degradation for controlled release. The microsphere system is based on degradation-controlled release, achieving sustained release for 1 to 6 months, suitable for late-stage local treatment, but it requires injection and is limited for patients with multiple joints in the early stage. The stimulus-responsive system (such as electricity, pH, temperature, enzymes) actively releases drugs on demand, with spatiotemporal control, avoiding systemic exposure, but the design is complex.

In summary, degradation-controlled release relies on the passive continuous release of material physical and chemical properties, with the release rate pre-set; stimulus-responsive uses environmental/external signals to actively trigger, improving targeting. Nanoparticles for targeted therapy, microspheres for long-term controlled release, and stimulus-responsive systems have complementary functions. They can be flexibly selected or combined according to the disease stage and requirements.

### 6.2 Direct regulation of immune cell phenotypes by scaffold and coating surfaces

The surface topological structure and biochemical signal regulation of the phenotypes of immune cells affect tissue repair and regeneration.

The micro-nano surface topography, such as the arrangement of nanofibers, influences the cytoskeleton through integrin α10β1/PI3K/AKT mechanical transduction. It can guide macrophages to polarize towards the repair-type M2 phenotype and upregulate the expression of anti-inflammatory factor IL-10 [137]. The 3D-printed β-TCP scaffold with negative Gaussian curvature (K < −1.72 mm^−2^) is sensed by macrophages, leading to microtubule destabilization and attenuation of the Ras-MAPK signaling pathway, which downregulates HIF-1α expression. This drives macrophage polarization toward the M2 phenotype. Subsequently, M2 macrophages secrete increased levels of pro-osteogenic and pro-angiogenic factors such as BMP-2, VEGF, and TGF-β, thereby achieving vascularization and bone regeneration [138]. Surface topography induces M2 polarization through mechanotransduction, secretes pro-angiogenic/osteogenic factors, and promotes repair and regeneration.

Continuous release of specific bioactive ions (such as Mo^6+^, Mg^2+^) can interfere with cellular metabolism. For instance, molybdenum ions can enhance the mitochondrial function of macrophages, promoting a shift in their metabolism from pro-inflammatory glycolysis to reparative oxidative phosphorylation, thereby stabilizing the M2 phenotype [139,140]. Mg^2+^ indirectly promotes osteogenesis and angiogenesis by regulating the local immune microenvironment [140]. Surface engineering grafting functional molecules such as glycosaminoglycan functional molecules and anti-fouling copolymers can directly transmit immunomodulatory signals or reduce fibrotic encapsulation. This significantly improves the biocompatibility and efficacy of implant materials [141,142]. Biochemical signals regulate metabolism and immune microenvironment, induce M2 polarization, reduce fibrosis, and improve repair and compatibility.

The specific size surface topological structure guides the M2 polarization of macrophages. The advantage lies in the stable signal and the absence of drug release, making it suitable for permanent or joint prosthesis and other long-term implants, with its own anti-inflammatory properties. The limitation is that the complex protein adsorption in the body may partially mask the regulatory effect, and the design parameters of size and shape need to match the target cell type.

Bioactive ion release schemes, such as Mg^2+^, Mo^6+^, achieve immune regulation by interfering with cellular metabolism. The advantage is that the ion signals can be dynamically sustained, and Mg^2+^ also has osteogenic and other repair functions. This scheme is most suitable for the coating of degradable filling scaffolds, and it can synergistically achieve immune regulation and tissue regeneration during the degradation process. However, the challenge lies in the precise control of release kinetics and concentration, and it is necessary to avoid toxicity or ineffective doses. Functional molecules grafted on the surface directly provide biochemical signals and achieve precise regulation. The advantage is high activity and strong specificity. The disadvantage is the unstable activity of the grafted molecules and potential immunogenicity. It is more suitable for the surface modification of short-term or medium-term degradable biomaterials. The three strategies are complementary, and they can be flexibly selected or combined according to the material type and treatment requirements.

### 6.3 Synergistic strategies of cell therapy and biomaterials

Biomaterials provide a biomimetic 3D microenvironment for therapeutic cells such as MSCs, resisting inflammatory erosion and enhancing their functions. The isolation-regulation strategy significantly improves the survival rate and targeting ability of cell therapy [143,144]. Biomaterials form a physical barrier, isolating the transplanted cells from the inflammatory environment. The mechanical properties of hydrogels, such as stiffness, are encoded as biochemical signals. The stiffness is perceived by cells through integrins, which then activate downstream mechanical signaling pathways. For example, the nuclear translocation of YAP and TAZ directly regulates the inflammatory phenotype of cells [145]. Biomedical materials can also serve as controlled-release repositories for cytokines. Covalently binding or physically encapsulating growth factors such as IL-4 within the scaffold enables sustained release. It continuously guides MSCs towards chondrocyte differentiation and synergistically regulates the local immune response with the secreted factors [146].

Physical isolation-regulatory materials provide a physical barrier that shields MSCs and other cells from inflammatory environments, improving cell survival and broad applicability, while their active regulatory capacity remains limited. Biomedical materials with mechanical encoding functions, such as regulating stiffness, can actively guide encapsulated cells and secretion by activating pathways like YAP/TAZ. Biomaterials can be upgraded from passive carriers to active guides, suitable for scenarios where cells need to perform specific repair procedures at the implant site. The disadvantage is that the design is complex and requires a deep understanding of the mechanical sensing pathways of the target cells. Growth factor controlled-release scaffolds combine the advantages of physical support and biochemical signal release and can temporally and spatially coordinate the guidance of cell behavior [147]. It is the most comprehensive solution, but has disadvantages such as poor stability of growth factors, difficulty in matching release curves, and high cost.

### 6.4 Active remodeling of the local joint immune microenvironment by biomaterials

The pathological core of joints with RA is the persistent presence of an inflammatory microenvironment. Intelligent materials can actively intervene through their physicochemical properties and reshape it into a reparative microenvironment. For instance, a thermosensitive hydrogel loaded with MgO nanoparticles gelates in the joint cavity and continuously releases Mg^2+^. It exerts anti-inflammatory effects by regulating macrophage polarization, as well as directly promotes angiogenesis and osteogenesis, thereby achieving the synergy of bone immune regulation and bone defect repair [140]. The degradation products of the materials themselves can also act as signaling molecules. For example, some polymer degradation products, such as butyrate esters, have known anti-inflammatory and histone deacetylase inhibitory activities, which can further inhibit the abnormal activation of local lymphocytes [69].

Intelligent responsive materials, pH or enzyme-responsive hydrogels, can release immunomodulators in response to the acidic and high enzyme activity triggers in joints with RA. Their greatest advantage is on-demand release, which improves the specificity and safety of the treatment and is suitable for the management of acute or fluctuating inflammatory periods with clear pathological markers. The limitation lies in their dependence on microenvironmental triggers. If the pathological signals are not strong, they may fail. Based on the degradation products of the materials themselves, such as certain polymers degrading to produce butyrate with anti-inflammatory activity, the advantage is that the therapeutic function is integrated with the metabolic process of the material without the need for additional drug loading, thus simplifying the system design. It is particularly suitable as a fully degradable implant or filler, providing long-term immunomodulatory effects through degradation products, while fulfilling the structural support function. However, the regulatory intensity and specificity are relatively fixed and external intervention or adjustment is difficult.

## 7 Conclusions and perspectives

In conclusion, the pathogenesis of RA is driven by the activation of immune cells and the disruption of immune homeostasis. The physicochemical properties of biomaterials, including surface chemistry, mechanical characteristics and morphological structure, enable the precise regulation of immune cell adhesion, polarization, activation and inflammatory cytokine secretion. This remodels the inflammatory microenvironment in RA. Thus, the rational design of these physicochemical parameters provides the theoretical basis and engineering strategies for achieving precise immunomodulation, providing new possibilities for RA therapy.

The immunomodulation of biomaterials for RA has shifted from a single anti-inflammatory effect to a coordinated regulation involving immunity, metabolism, and bone/cartilage regeneration. The immunomodulation and metabolic reprogramming of biomaterials interact with each other. Notably, M1 macrophages rely on glycolysis to promote inflammation, whereas M2 macrophages rely on oxidative phosphorylation [148]. Current studies are focused on designing biomaterials to carry the metabolic regulators lactate and α-ketoglutarate [149,150]. Lactate inhibits glycolysis and promotes M2 polarization at low concentrations or when ERK/STAT3 signaling pathways are activated through its bidirectional regulatory effects [149,151]. When T cells encounter hardened matrix in the RA synovium, they are activated through integrin-mediated mechanical stretching, which activates mTORC1 and promotes glycolysis and Th17 differentiation [152,153]. Simultaneously, stiffness affects Treg induction rate through different metabolisms. Metabolic responses should be considered in future material designs to achieve immune and metabolic synergy. Constructing scaffolds with variable stiffness can achieve sequential immune and metabolic remodeling.

The bone erosion and cartilage damage in RA are essentially immune dysregulation. Therefore, intelligent biomaterials regulate immunity to achieve tissue regeneration. Selenium-doped bioactive glass eliminates ROS, improves mitochondrial function, drives M2 polarization, and enhances bone regeneration [93]. Frontier designs construct temporal immune-regeneration scaffolds, such as the collagen-hyaluronic acid scaffold of MTX and miRNA-140, with early immunosuppression and later cartilage repair [76]. This transforms the treatment form of RA from symptom elimination to functional reconstruction.

Significant problems in current research and development persist. A contradiction exists between the single-factor dominance theory and the multi-parameter synergy effect. Some studies emphasize the dominant role of surface charge in macrophage polarization, whereas others emphasize the regulation of stiffness on T cell activation [154]. The primary reason for this contradiction is the simplification of biomaterial design, which ignores the influencing factors of other physical and chemical properties. When studying stiffness, a high stiffness of 295 kPa promotes the polarization of senescent macrophages to M1, whereas a low stiffness of 18–76 kPa promotes the polarization of anti-inflammatory M2 [64]. However, stiffness is not sensitive to other immune cells. The maturation state and antigen presentation efficiency of DCs are easily affected by surface functional groups, and T cells are sensitive to the elastic modulus and topological structure of biomaterials. This suggests that the various physical and chemical properties of biomaterials, such as charge, functional groups, and stiffness, do not act independently, but jointly shape the immune microenvironment through synergistic or antagonistic effects. For example, after hydrophilic chitosan is modified with hydrophobicity and coupled with FA, it can target the folic acid receptor of macrophages [50]. But if the stiffness or surface charge of the particles is changed simultaneously, the immunomodulatory effects remain to be systematically studied.

Numerous gaps still exist in the molecular mechanisms through which physical and chemical properties regulate immunity. For instance, material stiffness affects macrophage polarization through the YAP/TAZ pathway [28]. However, it remains unclear how YAP/TAZ influences the inflammatory signaling pathways, and how this mechanical signal transduction operates within the RA-specific high ROS and low pH microenvironment. Current studies focus on the effects of a single type of cell; however, the joint microenvironment of RA is a complex system involving the coordinated actions of multiple cells such as DCs, neutrophils, T cells, and B cells. For example, ROS-responsive nanoparticles can downregulate the CD40 expression of DCs and induce the differentiation of Tregs [41]. However, how this process affects the polarization direction of macrophages through paracrine or intercellular contact has not been systematically studied.

There are significant differences between the simplified in vitro models and the complex in vivo environment. Common 2D cultures cannot simulate the 3D ECM structure rich in collagen and hyaluronic acid in RA and the cell migration behavior. This leads to inconsistent conclusions between in vitro and in vivo studies. Simultaneously, the material characterization methods are disconnected from the in vivo dynamic monitoring. Traditional fluorescence labeling is difficult to track the degradation, phase transition and cell uptake processes of the material in deep tissues in real time and non-destructively, and new dynamic imaging techniques are urgently needed.

In view of these issues, future research should focus on the following aspects.

Firstly, a high-throughput, multi-parameter screening platform should be established. This standardized experimental system should incorporate cells derived from patients at different stages of the disease, including acute inflammation, erosion and chronic repair, as well as synovial fibroblasts and osteoclast precursors. Such a platform would enable the interactions among physicochemical parameters such as surface charge, stiffness, topography, and functional groups to be systematically evaluated and modelled.

Secondly, advanced single-cell omics and spatial transcriptomics should be integrated to characterize cellular states and spatial distributions across the synovium, pannus and bone erosion interfaces at various stages of the disease. These approaches will help to elucidate the dynamic molecular mechanisms underlying interactions between biomaterials and the immune microenvironment, particularly the convergence of mechanotransduction and inflammatory signaling networks.

Thirdly, patient-relevant in vitro models should be developed to recreate the 3D ECM architecture and pathological features of joints with RA, such as organoids and organ-on-a-chip platforms [[155], [156], [157], [158]]. These models should reproduce key pathological processes, including pannus formation and cartilage erosion. They should also incorporate cells derived from patients with heterogeneous autoantibody profiles and varying degrees of bone erosion to reflect individual variability [159,160]. In parallel, dynamic, non-invasive imaging and characterization methods are required to monitor material behavior and therapeutic responses in vivo.

Biomaterials with optimized physicochemical properties show great potential for the precise treatment of RA. However, RA is a highly dynamic and heterogeneous disease in terms of both its temporal progression and its manifestation in individual patients. In the acute phase, the disease is characterized primarily by synovial inflammation, immune cell infiltration and pannus formation. Therefore, biomaterials intended for this stage must respond rapidly to inflammatory cues, such as elevated ROS and low pH, to enable the on-demand release of anti-inflammatory agents. In the chronic phase, bone and cartilage erosion become the dominant pathological features. Consequently, biomaterials must provide adequate mechanical support, modulate immune responses, and promote bone and cartilage regeneration. Furthermore, patient heterogeneity, particularly regarding autoantibody profiles (such as ACPA and RF) and bone erosion scores, must inform material design. For instance, patients with high ACPA titers and severe bone erosion tend to have poorer prognoses, highlighting the need for stratified strategies. Composite scaffolds with enhanced osteogenic activity could be prioritized for patients at high risk of bone erosion, whereas responsive nanoformulations would be more suitable for patients with predominant inflammation. Accordingly, future material design should incorporate disease stage and patient subtype as key variables, rather than pursuing universal safety and stability.

In summary, future research should focus on RA disease staging and patient stratification. The aim should be to steer macrophage polarization from M1 to M2, correct aberrant immune cells, establish cross-scale evaluation systems and integrate materials science, immunology and clinical medicine. Ultimately, these efforts should result in targeted, responsive and self-regulating therapeutic platforms that can adapt dynamically to fluctuations in disease and align with individual pathological profiles.

## CRediT authorship contribution statement

**Rui Sun:** Writing – review & editing, Writing – original draft. **Chen Yue:** Project administration, Conceptualization. **Ning Yang:** Investigation, Conceptualization. **Ruoping Yanzhang:** Investigation, Conceptualization. **Peng Xiao:** Investigation, Conceptualization. **Youwen Liu:** Project administration, Conceptualization. **Xiaoqing Wang:** Project administration, Conceptualization. **Yiting Lei:** Writing – review & editing, Supervision, Project administration, Conceptualization. **Xiaolong Wu:** Project administration, Conceptualization. **Xue Zhang:** Writing – review & editing, Project administration, Conceptualization.

## Declaration of competing interest

The authors declare that they have no known competing financial interests or personal relationships that could have appeared to influence the work reported in this paper.

## Data Availability

No data was used for the research described in the article.
